# Associations between childhood maltreatment, PTSD and metabolic outcomes in patients with common mental disorders at outpatient clinics in specialized care

**DOI:** 10.1186/s12888-025-07346-6

**Published:** 2025-10-10

**Authors:** Steven Hoekstra, Miriam J. J. Lommen, Matthijs J. Warrens, Ellen Visser, Bennard Doornbos, Daniëlle Cath

**Affiliations:** 1 GGZ Drenthe Mental Health Institute, Department Trauma Center, Beilen, the Netherlands; 2 GGZ Drenthe Mental Health Institute, Department Adult Psychiatry, Assen, the Netherlands; 3 University of Groningen, Department of Clinical Psychology and Experimental Psychopathology, Grote Kruisstraat 2/1 9712 TS Groningen, the Netherlands; 4 University of Groningen, GION Education/Research, Groningen, the Netherlands; 5 University of Groningen, University Medical Center Groningen, University Center for Psychiatry, Rob Giel Research Center, Groningen, the Netherlands; 6 Lentis Research, Lentis Mental Health Institute, Groningen, the Netherlands

**Keywords:** PTSD, Childhood maltreatment, Metabolic syndrome

## Abstract

**Background:**

Childhood maltreatment is associated with an elevated risk of psychological disorders, including posttraumatic stress disorder (PTSD), but the impact on somatic consequences such as metabolic syndrome (MetS) is less well-known. This study aimed to examine these direct and indirect associations, and to exploratively assess whether these associations differ by sex.

**Methods:**

Somatic monitoring data of 528 outpatients (43.2% male; 56.8% female) were analysed. MetS was defined as the presence of at least three of five criteria: increased waist circumference, increased blood pressure, increased blood levels of triglycerides, increased blood levels of glucose, decreased blood levels of high-density lipoprotein (HDL-) cholesterol. Multiple regression analyses investigated the effects of childhood maltreatment severity directly and indirectly via PTSD symptom severity, relative to the influence of demographic and lifestyle related risk factors (age, sex, lifestyle-related behaviours, psychological distress, BMI and psychotropic medication use). Interaction terms between sex and childhood maltreatment severity and PTSD symptom severity were included. Moreover, two-way ANOVAs examined between-group differences of participants with or without presence of a history of childhood maltreatment and/or with or without presence of a PTSD.

**Results:**

Childhood maltreatment severity was directly related to increased waist circumference (2.115 [.06, 3.62]) and increased diastolic blood pressure (1.201 [0.23, 2.17]). Indirect associations between childhood maltreatment severity and metabolic outcomes through PTSD symptom severity were not significant, nor were the interaction terms. Indirect associations were found between lifestyle-related factors including smoking and unhealthy diet and MetS diagnosis and its components. Finally, higher blood glucose levels were found in participants with presence of a history of childhood maltreatment compared to those without.

**Conclusions:**

Childhood maltreatment is both directly and indirectly, via lifestyle-related factors, associated with components of MetS. Lifestyle-enhancing programs seem important to augment standard treatment for clinical populations, specifically with a history of childhood maltreatment, to reduce long-term somatic adversity.

**Trial registration:**

The MOPHAR research project has previously been registered with the Netherlands Trial Register on the 19th of November 2014 (NL4779). The research aspects of MOPHAR were approved by the independent medical ethics committee (RTPO 928, rTPO Leeuwarden, The Netherlands).

**Supplementary Information:**

The online version contains supplementary material available at 10.1186/s12888-025-07346-6.

## Background

Childhood maltreatment, defined as (emotional, physical and/or sexual) abuse and/or (emotional and/or physical) neglect in childhood and/or adolescence, has been identified as a risk factor for adverse outcomes. A review on Lifetime self-reported childhood maltreatment in Western samples revealed median prevalence rates of 20.4% to 28.8% for sexual abuse, 12.0% to 27.0% for physical abuse, 6.2% to 28.4% for emotional abuse, and 16.6% to 40.5% for emotional/physical neglect. Overall, girls are at a higher risk for sexual, physical, and emotional abuse, as well as neglect [1]. In addition, a review of studies among adult patients with anxiety or depressive disorder in the Netherlands revealed that up to forty percent self-reported at least one type of childhood maltreatment. Rates ranged between 13.8% and 38.9%, with the lowest for physical abuse and the highest for emotional neglect [2].

The impact of childhood maltreatment on adult psychological consequences has been extensively researched. Meta-analyses have shown associations with adolescence and adult psychiatric disorders, including depression [3], anxiety disorders [4], suicidality and suicidal ideation [5], non-suicidal self-injury [6] and substance use disorder [7]. Furthermore, childhood maltreatment was linked to trauma-related pathology, including posttraumatic stress disorder (PTSD) [8] and dissociation [9]. The somatic consequences of childhood maltreatment are also becoming more widely recognised. In a scoping review of 40 studies, 91.7% reported positive associations between childhood maltreatment and cardiovascular disease (CVD), 88.2% reported positive associations with type 2 diabetes mellitus, and 61.5% reported positive associations with elevated blood pressure. The inclusion of (multiple) mental disorders, including depression, anxiety disorders and/or PTSD weakened the associations, but childhood maltreatment was still significantly associated in almost all cases [10].

Metabolic syndrome (MetS), as defined by the Adult Treatment Panel III (ATP-III), was introduced to assist clinicians in identifying patients at increased risk of developing CVD [11]. A diagnosis of MetS requires the presence of at least three of five components. These include increased waist circumference (> 102 cm for men and > 88 cm for women), elevated blood pressure (systolic ≥ 130 mmHg or diastolic ≥ 85 mmHg), increased blood levels of triglycerides (≥ 1.7 mmol/L), increased blood levels of glucose (fasting ≥ 6.1 mmol/L, or non-fasting glucose ≥ 7.8 mmol/L), or decreased blood levels of high-density lipoprotein (HDL-) cholesterol (< 1.0 mmol/L for men and < 1.3 mmol/L for women). A strong association has been found between MetS and CVD [12]. The somatic risk factors that reflect the components of MetS are prevalent amongst those who report a history of childhood maltreatment [13], with a 40% increased risk of adult cardiometabolic disease, type 2 diabetes mellitus and MetS in individuals who experienced childhood maltreatment compared to those without childhood maltreatment [13].

In addition, PTSD is linked to an increased likelihood of developing adverse somatic consequences [10, 14], with prevalence rates of MetS ranging from 31.9% to 47.8% [15]. A two- to three-fold mortality rate was observed in individuals with PTSD compared to the general population [16] and sex/age-matched controls [14], with most cases showing elevated abdominal obesity, blood pressure, and blood levels of triglycerides and decreased blood levels of HDL-cholesterol [14]. The findings of increased risk for somatic consequences of PTSD were replicated in a more recent retrospective, register-based study with a 6-year follow-up of adult PTSD patients (*n* = 7,852) of the general Norwegian population (*n* = 4,041,366). The study revealed higher hazard ratios (HR) for a range of cardiometabolic diseases among PTSD patients, including hypertensive diseases (HR: 3.5, 99% CI: 3.1–3.9) and obesity (HR: 6.5, 99% CI: 5.7–7.5). Adjustment for socioeconomic status and comorbid mental disorders, especially depression, resulted in HR reductions of 48.6% and 67.7%, respectively [17].

Interestingly, the majority of included studies in the aforementioned meta-analysis on the prevalence of MetS by Rosenbaum and colleagues (2015) [14] were based on studies in veterans with war-related PTSD. The analysis only included three studies focusing on subjects with non-war related trauma, comprising 203 outpatients [18], 115 police officers [19] and 351 repatriated prisoners of war [20]. This highlights a key Limitation in the current Literature, namely the underrepresentation of populations with PTSD with non-war related causes, and further reveals that few studies have directly investigated the specific impact of childhood maltreatment on physical health outcomes such as MetS. To the best of our knowledge, only one study investigated the impact of childhood maltreatment and PTSD on MetS in concert in 337 veterans of war of whom 121 had experienced early life trauma [21]. This study revealed that this group is at a threefold increased risk of developing metabolic abnormalities compared to those without such a history [21].

There is compelling evidence that the prevalence of MetS differs between men and women and changes with age due to social, cultural and biological factors [13, 22]. A large European study (*n* = 69,094) demonstrated that MetS prevalence increases fivefold in women from the ages of 19–39 to 60–78 years, compared to a twofold increase in men. This reflects a notably smaller rise in men over 49 years and may be explained by postmenopausal increases in blood pressure in women [23]. In a French sample (*n* = 3,508), elevated blood pressure was a significant contributor to MetS in men, while elevated body mass index (BMI), elevated waist circumference, and decreased blood levels of HDL-cholesterol were significant contributors in women [24].

Similar sex-related patterns in relation to MetS have been observed in individuals diagnosed with PTSD in Chinese (*n* = 2,687) [25], Taiwanese (*n* = 953) [26], and South Korean samples (*n* = 107) [27]. However, conflicting results have been observed as well, since a study in 1,355 U.S. veterans found no evidence that PTSD was differentially associated with MetS as a function of sex [28]. The few studies that have examined sex-specific differences in the association between MetS and childhood maltreatment, found that emotional, physical and cumulative adversity elevated the metabolic risk for both men (*n* = 534) and women (*n* = 700), while sexual abuse increased risk only in women [29].

The objective of this study is to replicate and extend existing literature by examining the relation between childhood maltreatment, PTSD, and (components of) MetS in ‘real life’ patients referred to secondary psychiatric care while including lifestyle-related and demographic known risk factors of MetS in the analyses. We formulated the following research questions: (1) What is the direct association between childhood maltreatment severity and MetS and its individual components in adults with psychiatric disorders? (2) What is the indirect association of childhood maltreatment severity through PTSD symptom severity in adulthood relative to other known demographic and lifestyle related risk factors of MetS (e.g., age, sex, lifestyle-related behaviours, psychological distress, BMI and use of psychotropic medication)? (3). Research question 3 was exploratory in nature and examined whether sex moderates the associations between childhood maltreatment severity, PTSD symptom severity, and MetS. Accordingly, no specific hypothesis was proposed. (4). What are the differences between somatic outcomes in participants with or without presence of a history of childhood maltreatment and participants with or without presence of a PTSD?

We hypothesised that (1) severity of childhood maltreatment would be directly associated with poorer metabolic outcomes, including the presence of MetS and its constituent components. (2) We hypothesised an indirect effect of childhood maltreatment severity on poorer metabolic outcomes through PTSD symptom severity, even after adjusting for other known demographic and lifestyle related risk factors of MetS. (4) We hypothesised that participants with both presence of a history of childhood maltreatment and a PTSD would show the highest prevalence of MetS and its constituent components.

## Method

### Study group and procedures

The present study used a cross-sectional design, based on baseline data from the MOPHAR study (Monitoring Outcomes of Psychopharmacotherapy) [30], a somatic monitoring program conducted in specialized mental health outpatient clinics in the Northern Netherlands. Although MOPHAR includes repeated measurements (at intake and approximately 12 months later), only the data collected at the initial assessment were used for the current analyses.

All patients, irrespective of the nature of their psychological complaints, are invited to participate in the program. MOPHAR is designed to perform medication reconciliation, and to detect somatic risk factors, somatic comorbidities and subjective as well as objective (side-) effects of psychotropic medication. A brief somatic screening is conducted by trained nurses, comprising measurements of length, weight, blood pressure, and waist circumference. Patients are interviewed on smoking behaviour, alcohol- and drug use, and other lifestyle-related behaviours. Laboratory examinations are performed on, among others, blood levels of HDL-cholesterol, triglycerides and glucose. Furthermore, the program incorporates online self-reported routine outcome monitoring of psychological symptoms.

After the provision of verbal and written information, all participants provided informed consent for the utilisation of anonymized data. The MOPHAR research project has previously been registered with the Netherlands Trial Register on the 19th of November 2014 (NL4779). The research aspects of MOPHAR were approved by the independent medical ethics committee (RTPO 928, rTPO Leeuwarden, The Netherlands). For a comprehensive description of the MOPHAR program, see [31].

From 2020 onwards, the MOPHAR program was expanded to include self-report measures of PTSD and childhood maltreatment. The current study has been carried out on a subsample of participants with information available on these variables (*n* = 528). To ascertain the representativeness of this sample relative to the full MOPHAR patient sample collected between 2016 and 2021 (*n* = 1,897), comparative analyses between the two samples were conducted on sex, age and the five dichotomised MetS components by inspecting group means and frequencies. Since no substantial differences were observed in these descriptive comparisons, it was assumed that our subsample, with information available on the self-report measures of PTSD and childhood maltreatment, is representative for the full MOPHAR sample in whom there was no information available on these variables (see Appendix A).

### Instruments

#### Childhood maltreatment

Childhood maltreatment was measured with the Dutch translation of the Childhood Trauma Questionnaire-Short Form (CTQ-SF) [32, 33]. The CTQ-SF is designed to evaluate adverse experiences that occurred prior to the age of 15 years. The instrument entails five subscales, each comprising five items, rated on a Likert scale ranging from 1 ‘*Never true*’ to 5 ‘*Very often true*’ (range 5–25).

To answer research questions 1, 2 and 3, childhood maltreatment severity was calculated as the total sum score of the CTQ-SF (range 0–125). To answer research question 4, each CTQ-SF subscale score was dichotomized base on established thresholds for ‘low to moderate’ severity (emotional abuse ≥ 9, physical abuse ≥ 8, sexual abuse ≥ 6, emotional neglect ≥ 10, and physical neglect ≥ 8). For each subscale, a score meeting or exceeding the threshold was coded as present, and a score below the threshold as absent. These binary indicators were then summed across the five subscales, reflecting the number of maltreatment subtypes experienced. Childhood maltreatment was considered present if a participant reported at least one subscale. The Cronbach’s alpha’s (α) of the subscales in the present sample was consistent with those observed in previous studies [34], with a range of α-values between 0.72 and 0.95.

#### PTSD

PTSD was measured with the Dutch version of the PTSD Checklist for the Diagnostic and Statistical Manual of Mental Disorders, 5th edition (PCL-5) [35], because we were unable to verify the correctness of the clinician-derived PTSD diagnoses. The PCL-5 entails 20 statements, rated on a scale of 0 ‘*Not at all*’ to 4 ‘*Extremely*’ (range 0–80). Higher sum scores indicate greater PTSD symptom severity experienced in the past month. To answer research questions 1, 2 and 3, PTSD symptom severity was calculated as the total sum score of PCL-5. To answer research question 4, a probable diagnosis of PTSD was made using cut-off scores ≥ 33 [36]. The PCL-5 has demonstrated robust psychometric properties in previous studies [37]. Cronbach’s α in the present study was 0.93.

#### Demographic and lifestyle related risk factors

Other demographic and lifestyle related risk factors for MetS and its constituent components included alcohol use, smoking, healthy/unhealthy diet, global disability, psychological distress, BMI, psychotropic medication use, age and sex.

Alcohol use over the past three months was measured with the Dutch version of the Alcohol Use Disorders Identification Test (AUDIT) [38, 39], in which 10 items were scored on a 0 ‘*Never*’ to 4 ‘*4 or more times a week*’ Likert scale (range 0–40). Higher scores indicate more problematic alcohol use, as indicated by more alcohol consumption, drinking behaviours and alcohol-related problems. Smoking was evaluated through a multiple-choice question, assessing the consumption of various nicotine-containing substances including cigarettes, rolling tobacco, cigars, pipes or other forms of tobacco. The participants were asked to indicate whether they had ever smoked, were currently smoking, or had never smoked. Current or past smokers were asked to quantify the number of nicotine-containing substances they consume(d) on a daily basis. Dietary habits were measured with three questions pertaining to the consumption of fruits (less than three per day), vegetables (less than four times a week) and ready-made meals (more than two days a week). Higher scores (range 0–3) indicated less healthy dietary patterns. Global disability was measured with the World Health Organization Disability Assessment Schedule (WHO-DAS 2.0), which includes 12 items scored on a 1 ‘*None*’ to 5 ‘*Extreme or cannot do*’ Likert scale [40], and indicated to which extent health issues impeded ability to engage in daily activities over the past 30 days (range 12–60). Higher scores indicate more constraints [41]. Psychological distress was measured with the Dutch version of the Outcome Questionnaire-45 (OQ-45) [42, 43] with items scored on a 0 ‘*Never*’ to 4 ‘*Almost always*’ Likert scale. In the current study, only the 25-item Symptomatic Distress subscale was employed (range 0–100), with higher scores indicating a higher severity of depressive, stress and anxiety symptoms. Cronbach’s α of the AUDIT, WHO-DAS 2.0 and OQ-45 ranged between 0.85 and 0.89.

BMI was calculated by dividing the participants’ weight (in kilograms) by their squared height (in meters). The current use of antipsychotics, antidepressants and mood stabilisers was operationalised as a numerical value representing the number of psychotropic medication used (range 0–3). Sex was assessed by a multiple-choice question (male, female, other).

#### Metabolic syndrome

The presence of MetS was determined primarily according to ATP-III criteria [11]. In accordance with this definition, the presence of at least three of the following five components is required: waist circumference (> 102 cm for men and > 88 cm for women), blood pressure (systolic ≥ 130 mmHg or diastolic ≥ 85 mmHg), blood levels of HDL-cholesterol (< 1.0 mmol/L for men and < 1.3 mmol/L for women), blood levels of triglycerides (≥ 1.7 mmol/L), and blood levels of glucose (fasting ≥ 6.1 mmol/L, or non-fasting glucose ≥ 7.8 mmol/L) [44]. Cut-off values for fasting and non-fasting glucose were based on Dutch general practitioners’ guideline [45]. In the analysis with blood glucose levels as outcome, the fasting and non-fasting glucose results were combined by adding 1.7 mmol/L to the fasting-glucose values. In the event that participants were taking medication for the components (e.g., lipid-lowering, antihypertensive, and/or anti-diabetic medication), their criterion for that respective MetS component was deemed to be present.

### Data-analysis

All analyses were conducted using IBM SPSS Statistics 28 software. All non-dichotomous variables were standardised into z-scores prior to the analyses. For all tests, a significance level of 0.05 was employed. Only tables describing the study group characteristics and providing information on the main hypotheses are included in the manuscript, additional analyses are presented in the appendices. For further details regarding the data cleaning process and assumptions check, see Appendix B, E and F.

The present study incorporated both dimensional (to address research questions 1, 2 and 3) and binary (to address research question 4) operationalisations of childhood maltreatment and PTSD. The binary variables enabled the examination of potential threshold effects in a clinically interpretable manner, while the dimensional score facilitated the assessment of more nuanced ‘dose’–response relationships. This mixed approach was intended to balance statistical sensitivity with clinical relevance and interpretability.

To test the first research question, that childhood maltreatment severity would be directly associated with poorer metabolic outcomes, regression analyses were conducted with the continuous MetS components (e.g., waist circumference, systolic and diastolic blood pressure, blood levels of HDL-cholesterol, triglycerides and glucose) as outcome variables and childhood maltreatment severity as independent variable. A separate model was estimated for each outcome variable. Additionally, a multiple logistic regression analysis was conducted with the presence/absence of a MetS diagnosis as the outcome variable (Models 1).

To test the second research question, that childhood maltreatment would be indirectly associated with poorer metabolic outcomes through PTSD symptom severity, PTSD symptom severity and other known demographic and lifestyle related risk factors for MetS (e.g., age, sex, lifestyle-related behaviours, psychological distress, BMI and use of psychotropic medication) were added to Models 1 to explore potential indirect pathways (Models 2).

To test the third exploratory research question, whether sex moderates the associations between childhood maltreatment severity, PTSD symptom severity, and MetS, we included interaction terms between sex and childhood maltreatment severity, and between sex and PTSD symptom severity in Models 2*.*.

To test the fourth research question, that participants with both presence of a childhood maltreatment history and presence of a PTSD (both as defined here-above) would show the highest prevalence of MetS and its constituent components, two-way ANOVAs were conducted, with the continuous MetS components as the dependent variables. A logistic regression analysis was conducted with MetS diagnosis as a dependent variable. Post-hoc comparisons with Bonferroni correction were conducted when significant differences were found between the four groups based on the ANOVA results. Bonferroni corrections were employed to deal with multiple comparisons (0.05/6).

The use of medication for the treatment of somatic adversities (e.g., lipid-lowering, antihypertensive, and anti-diabetic medication) constitutes a defining component of a MetS diagnosis and, as a result, was not included in the analysis where a MetS diagnosis was the outcome variable. However, they were included in the analyses where the continuous individual MetS components were the outcome variables. Considering the established high correlations between BMI and waist circumference, BMI was excluded as a covariate in the analysis with waist circumference as the outcome variable to avoid multicollinearity. Given that data collection was partially conducted during the Covid-19 pandemic, with many participants reluctant to have anthropomorphic measurements and blood draws at the laboratory, the resulting sample sizes varied from *n* = 178 to *n* = 509 for Models 1 and from *n* = 82 to *n* = 250 for Models 2. To ascertain the representativeness of the sample with versus without information on dichotomised MetS and its constituent components, the groups were descriptively compared on the mean PCL-5 scores, severity of childhood maltreatment scores (0–100), and demographic and lifestyle related risk factors (see Appendix C). As no meaningful differences were observed in blood pressure and blood levels of HDL-cholesterol, triglycerides and glucose on these variables, it was assumed that data for these variables were missing at random. Participants with data on waist circumference were, on average, younger, exhibited lower BMI and were more likely to engage in smoking and alcohol use compared to those without.

## Results

### Total sample

#### Demographic and clinical characteristics

The mean age of the sample (*n* = 528) was 37.83 years (SD = 11.90). A total of 228 participants (43.2%) were male and 300 participants (56.8%) were female (see Table 1). Of the sample, 72 participants (13.6%) scored below the threshold for experienced childhood maltreatment, 103 participants (19.5%) scored above the threshold on one type of childhood maltreatment, and 118 participants (22.3%) on two, 107 participants (20.3%) on three, 68 participants (12.9%) on four and 60 participants (11.4%) scored on all five subtypes. A total of 308 participants (58.3%) met criteria of a probable diagnosis of PTSD. A total of 181 participants (34.3%) reported childhood maltreatment only, 33 participants (6.3%) had a probable diagnosis of PTSD only, 275 participants (52.1%) reported both and 39 (7.4%) reported neither.

**Table 1:** Demographic and clinical characteristics of the sample and male and female subsample

		*n*	Mean (SD)			
Demographic information			Total	Male	Female	
Sex		528	-	228 (43.2%)	300 (56.8%)	
Age		527	37.83 (11.90)	39.81 (12.01)	36.32 (11.59)*	
Clinical characteristics						
Variable	Subscale	*n*	Mean (SD)	Above cut-off		
			Total	Total	Male	Female
PTSD		528	36.22 (16.8)	308 (58.3%)	122 (53.5%)	186 (62.0%)
Childhood maltreatment	Emotional abuse	528	10.90 (5.4)	304 (57.6%)	120 (52.6%)	184 (61.3%)^*^
	Physical abuse	528	6.89 (3.8)	114 (21.6%)	50 (21.9%)	64 (21.3%)
	Sexual abuse	528	7.43 (5.0)	165 (31.3%)	40 (17.5%)	125 (41.7%)^**^
	Emotional neglect	528	14.27 (5.23)	417 (79.0%)	179 (78.5%)	238 (79.3%)
	Physical neglect	528	8.07 (3.4)	232 (43.9%)	99 (43.4%)	133 (44.3%)
Somatic	Waist circumference	509	97.64 (17.3)	279 (52.8%)	105 (47.7%)	174 (60.2%)^**^
	Blood pressure	-	-	-	-	-
	- Systolic	304	128.96 (18.2)	156 (51.3%)	88 (70.4%)	68 (38.0%)^**^
	- Diastolic	304	82.37 (11.2)	152 (50%)	72 (57.6%)	80 (44.7%)^*^
	Blood levels of HDL-cholesterol	139	1.34 (.40)	67 (48.2%)	32 (52.5%)	35 (44.9%)
	Blood levels of triglycerides	139	1.48 (.90)	57 (41.0%)	32 (52.5%)	25 (32.1%)^*^
	Blood levels of glucose	197	6.24 (1.4)	16 (7.1%)	9 (8.1%)	7 (5.4%)
	Metabolic syndrome	178	N/A	59 (33.1%)	31 (43.7%)	28 (26.2%)^*^
	BMI	511	27.24 (6.2)	303 (59.4%)	142 (63.7%)	165 (57.3%)
	Number of psychotropic medication	314	.69 (.70)	-	-	
Psychological distress	Psychological distress	497	45.71 (13.7)	410 (82.5%)	172 (80.0%)	238 (84.4%)
Global disability	-	528	26.45 (9.0)^c^	-	-	-
Smoking	-	486	8.06 (9.9)^b^	274 (56.4%)	117 (55.7%)	157 (56.9%)
Alcohol use	-	474	3.76 (5.1)	67 (14.1%)	44 (21.5%)	23 (8.6%)^**^
Healthy/unhealthy diet	-	527	1.13 (.6)^c^	-	-	-

Childhood maltreatment and PTSD were assessed using the CTQ-SF and PCL-5, respectively. Alcohol use was assessed using the AUDIT, which measures alcohol consumption, drinking behaviours, and alcohol-related problems. Global disability was measured with the WHO-DAS 2.0, indicating the extent to which health issues interfered with daily functioning in the past 30 days. Psychological distress reflects scores on the OQ-45 Symptomatic Distress subscale, capturing symptoms of depression, stress, and anxiety. *p* <.005*, *p* <.001**. ^a^ = sum score of the childhood maltreatment present or absent subtypes (range 0–5); ^b^ = mean number of nicotine-containing substances; ^c^ = in the absence of established cut-off scores, the variables were compared on the basis of significant differences in mean scores between men and women. No statistically differences were identified

Of all participants, 178 (33.1%) had sufficient information on individual MetS components to establish a MetS diagnosis. Of these participants, 15 (8.4%) exhibited no MetS components at baseline, 70 participants (39.3%) demonstrated one component, 34 (19.1%) two components, and 59 (33.1%) exhibited three or more components.

#### Associations of childhood maltreatment with metabolic outcomes

Regarding direct effects of childhood maltreatment on metabolic outcomes, significant associations were observed with waist circumference (2.115 [0.06, 3.62]) and diastolic blood pressure (1.201 [0.23, 2.17]).

#### Associations of PTSD with metabolic outcomes

PTSD symptom severity was not found to be significantly associated with any of the individual MetS components (see Table 2). In Table 2*,* only (un)standardized beta-coefficients of childhood maltreatment severity, PTSD symptom severity, and their interactions with sex are visualized to enhance readability. However, all lifestyle-related and demographic risk factors and the interaction terms were included simultaneously. For a detailed exposition of these results, see Appendix D. Overall model tests are presented in Appendix B.

**Table 2:** Results of multiple (logistic) regression analyses

Dependent variable	**Waist circumference**^**a**^				**Metabolic syndrome**^**b**^			
	**Model 1** adjusted *R*^*2*^ = *.*013		**Model 2** adjusted *R*^*2*^ = *.*172		***Model 1*** Nagelkerke’s *R*^*2*^ =.004		***Model 2*** Nagelkerke’s *R*^*2*^ =.427	
	***B [95% CI]***	*β*	***B [95% CI]***	*β*	***B [95% CI]***	**OR *[95% CI]***	***B [95% CI]***	OR ***[95% CI]***
**Constant**	97.639 [96.14, 99.13]**		104.024 [99.89, 108.15]**		-.717 [−1.03, -.40]**	.488 [.36,.67]	.046 [-.84,.93]	1.047 [.43, 2.54]
**Childhood maltreatment**	2.115 [.060, 3.62]**	.121	1.384 [−1.98, 4.75]	.084	.119 [-.20,.43]	1.126 [.82, 1.54]	.223 [-.69, 1.14]	1.250 [.50, 3.13]
**PTSD symptom severity**			2.131 [−2.12, 6.38]	.121			.342 [-.70, 1.38]	1.407 [.50, 3.98]
**Childhood maltreatment severity x sex**			-.716 [−5.11, 3.68]	-.033			-.198 [−1.41, 1.01]	.821 [.68, 7.60]
**PTSD symptom severity x sex**			.303 [−4.43, 5.04]	.013			549 [-.071, 1.81]	1.732 [1.61, 19.85]
	**Systolic blood pressure**^**a**^				**Diastolic blood pressure**^**a**^			
	**Model 1** adjusted *R*^*2*^ = -.001		**Model 2** adjusted *R*^*2*^ = *.*332		**Model 1** adjusted *R*^*2*^ = *.*010		**Model 2** adjusted *R*^*2*^ = *.*232	
	***B [95% CI]***	*β*	***B [95% CI]***	*β*	***B [95% CI]***	*β*	***B [95% CI]***	*β*
**Constant**	128.957 [127.37, 130.54]**		136.849 [132.85, 140.84]**		82.364 [81.39, 83.34]**		86.331[83.93, 88.73]**	
**Childhood maltreatment**	.509 [−1.07, 2.09]	-.028	.220 [−3.04, 3.48]	.013	1.201 [0.23, 2.17]*	.107	1.305 [-.66, 3.26]	.133
**PTSD symptom severity**			.184 [−3.85, 4.21]	.010			−1.507 [−3.93,.92]	-.100
**Childhood maltreatment severity x sex**			-.806 [−4.99, 3.38]	-.036			−1.152 [−3.67, 1.36]	-.092
**PTSD symptom severity x sex**			-.270 [−4.78, 4.24]	-.011			.879 [−1.83, 3.59]	.064
	**Blood levels of HDL-cholesterol**^**a**^				**Blood levels of triglycerides**^**a**^			
	**Model 1** adjusted *R*^*2*^ = -.001		**Model 2** adjusted *R*^*2*^ = *.*169		**Model 1** adjusted *R*^*2*^ = *.*006		**Model 2** adjusted *R*^*2*^ = *.*313	
	***B [95% CI]***	*β*	***B [95% CI]***	*β*	***B [95% CI]***	*β*	***B [95% CI]***	*β*
**Constant**	1.342 [1.29, 1.39]**		1.276 [1.13, 1.42]**		1.477 [1.37, 1.59]**		1.768 [1.44, 2.10]**	
**Childhood maltreatment**	-.024 [−0.07, 0.03]	-.058	-.012 [-.14,.12]	-.031	.093 [-.02,.021]	.097	.187 [-.11,.48]	.199
**PTSD symptom severity**			-.076 [-.22,.07]	-.209			.038 [-.29,.36]	.042
**Childhood maltreatment severity x sex**			.080 [-.08,.24]	.157			-.329 [-.70,.04]	-.259
**PTSD symptom severity x sex**			.009 [-.15,.17]	.018			.053 [-.31, 0.42]	.041
	**Blood levels of glucose**^**a**^							
	**Model 1** adjusted *R*^*2*^ = -.001		**Model 2** adjusted *R*^*2*^ = *.*125					
	***B [95% CI]***	*β*	***B [95% CI]***	*β*				
**Constant**	6.420 [6.23, 6.61]**		6.582 [6.14, 7.02]**					
**Childhood maltreatment**	.096 [-.10,.30]	.068	-.137 [-.52,.25]	-.146				
**PTSD symptom severity**			.083 [-.32,.48]	.093				
**Childhood maltreatment severity x sex**			.181 [-.29,.66]	.138				
**PTSD symptom severity x sex**			-.290 [-.74,.16]	-.221				

Childhood maltreatment severity and PTSD symptom severity were assessed using the CTQ-SF and PCL-5, respectively. In the analyses, men were coded as 0 and women as 1. ***B*** = unstandardized beta-coefficient; *β* = standardized beta-coefficient; CI = confidence interval; *p* <.005*, *p* <.001**. All predictors were standardized (z-scores) prior to analysis. The interaction term represents the product of the standardized variables. In this table*, *only (un)standardized beta-coefficients of childhood maltreatment severity, PTSD symptom severity, and their interactions with sex are visualized. However, all known risk factors were included in Models 2. For these results, see Appendix D*. ****B*** = unstandardized beta-coefficient; *β* = standardized beta-coefficient; OR = odds ratio; ^a^ multiple regression; ^b^ multiple logistic regression

The regression coefficients of the demographic and lifestyle-related risk factors on MetS and its individual components were relatively large compared to the small and mostly non-significant coefficients of childhood maltreatment on MetS. Healthy/unhealthy diet (3.240 [1.01, 5.47]), antihypertensive use (5.797 [0.45, 11.14]), age (6.523 [3.60, 9.45]) and being female (5.652 [−9.91, −1.40]) were significantly associated with waist circumference. BMI (6.149 [4.31, 7.99]), age (5.329 [2.47, 8.19]) and being male (−10.754[−14.88, −6.63) were significantly associated with systolic blood pressure, and BMI (3.175 [2.07, 4.280]) and age (4.108[2.39, 5.83]) with diastolic blood pressure. Moreover, BMI (−0.061 [−0.12, −0.01]) and being male (0.278 [0.14, 0.42]) were associated with blood levels of HDL-cholesterol, smoking (309 [0.13, 0.49]), BMI (225 [0.10, 0.35]) and being male (−0.475 [−0.79, −0.16]) with blood levels of triglycerides, and statins (0.886 [0.06, 1.71]) and age (0.474 [0.18, 0.77]) with blood levels of glucose. Lastly, BMI (1.031 [0.48, 1.58]) and being male (−1.431 [−2.52, −0.35]) were significantly related to MetS (see Appendix D). For a summary, see Appendix B.

### Male and female subsample

#### Demographic and clinical characteristics

The women in the current sample were on average significantly younger than the men (36.32 (11.59 [18–63]), versus 39.81 (12.01 [18–63]). Both male and female participants exhibited high percentages above the cut-off scores for childhood neglect/abuse, with 87% of women versus 85.5% of men reporting one or more types of maltreatment. Specifically, more women reported a history of sexual abuse (41.7% versus 17.5% in men) and emotional abuse (61.3% versus 52.6% in men).

Both a MetS diagnosis and increased levels of its components were more often found in men than in women, with increased systolic (70.4% in men versus 38.0% in women) and diastolic (57.6% in men versus 44.7% in women) blood pressure, higher proportions of elevated blood levels of triglycerides (52.5% in men versus 44.9% in women) and of MetS diagnosis (43.7% in men versus 26.2% in women).

Regarding demographic and lifestyle related risk factors, women were significantly younger than men (36.32 versus 39.81) and the proportion of individuals engaging in problematic alcohol use was significantly higher among men than women (21.5% versus 8.6%).

#### Associations of childhood maltreatment and PTSD with metabolic outcomes

No statistically significant sex-specific interaction effects were found in the associations between childhood maltreatment severity, PTSD symptom severity and any of the individual MetS components, or a MetS diagnosis (see Table 2).

### Somatic outcomes across groups with or without childhood maltreatment and with or without PTSD

With regard to the individual MetS components, participants who reported presence of a history of childhood maltreatment exhibited higher mean blood levels of glucose than those without a history of childhood maltreatment (see Table 3). No other between-group differences were identified at the 0.05 level nor the 0.05/6 level with respect to the individual MetS components. No significant between-group difference was found on MetS diagnosis (results not shown).

**Table 3:** Results of two-way ANOVA analyses

	**Waist circumference**		**Systolic blood pressure**		**Diastolic blood pressure**		**Blood levels of HDL-cholesterol**			**Blood levels of triglycerides**			**Blood levels of glucose**	
	**MD**	***p***	**MD**	***p***	**MD**	***p***	**MD**	***p***		**MD**		***p***	**MD**	***p***
**PTSD present/absent**	2.96	.117	.08	.567	.94	.336	.0013	.961		.1913		.091	.1221	.751
**Childhood maltreatment present/absent**	1.42	.652	1.99	.405	2.29	.135	.1006	.183		.0083		.864	.7224	.020*
**Interaction-effect**		.700		.404		.556		.901				.442		.504

*BP* blood pressure, *HDL-C* HDL-cholesterol, *MD* mean difference

*p* <.005*

With respect to the demographic and lifestyle-related risk factors, significant overall group differences were observed with regard to BMI, smoking behaviour, global disability, and psychological distress (see Table 4). Post-hoc comparisons revealed that participants with both PTSD and childhood maltreatment smoked more, had a higher BMI than those with childhood maltreatment only, and reported significantly higher psychological distress and global disability scores than all other groups. Additionally, participants with PTSD only scored higher than those with childhood maltreatment only on both psychological distress and global disability (data not shown).

**Table 4:** Demographic and clinical characteristics per group with/without a history of childhood maltreatment and with/without PTSD

Variable	Subscale		Mean (SD)		Mean (SD)		Mean (SD)		Mean (SD)
		*n*	No childhood maltreatment, nor PTSD	*n*	PTSD only	*n*	Childhood maltreatment only	*n*	Childhood maltreatment and PTSD
		Men	19 (48.7%)		14 (42.4%)		87 (48.1%)		108 (39.3%)
		Women	20 (51.3%)		19 (57.6%)		94 (51.9%)		167 (60.7%)
		Age total	36.54 (12.5)		39.18 (13.7)		37.45 (11.8)		38.11 (11.7)
PTSD		39	18.21 (10.3)	33	45.12 (8.0)	181	20.07 (8.4)	275	48.33 (9.9)
Childhood maltreatment	Emotional abuse	39	5.79 (1.0)	33	5.76 (1.1)	181	9.46 (3.9)	275	13.18 (5.7)
	Physical abuse	39	5.00 (.0)	33	5.06 (.4)	181	6.14 (2.6)	275	7.87 (4.6)
	Sexual abuse	39	5.00 (.0)	33	5.00 (.0)	181	6.29 (3.1)	275	8.82 (6.1)
	Emotional neglect	39	7.08 (1.4)	33	6.94 (.2)	181	14.19 (4.2)	275	16.23 (4.8)
	Physical neglect	39	5.26 (.6)	33	5.18 (.5)	181	7.64 (2.7)	275	9.09 (3.8)
	Severity (0–5)^a^	39	.00 (.0)	33	.00 (.0)	181	2.24 (1.1)	275	3.01 (1.4)
Somatic	Waist circumference	37	94.35 (14.1)	33	98.73 (17.0)	173	96.23 (18.0)	266	98.88 (17.3)
	Blood pressure								
	- Systolic	37	125.689 (16.3)	33	129 (22.8)	170	129.61 (15.6)	268	128.99 (19.4)
	- Diastolic	37	79.32 (10.1)	33	81.58 (11.6)	170	82.35 (11.0)	268	82.90 (11.5)
	Blood levels of HDL-cholesterol	16	1.25 (.3)	15	1.26 (.4)	93	1.36 (.4)	134	1.35 (.4)
	Blood levels of triglycerides	16	1.28 (.8)	15	1.70 (1.1)	93	1.38 (.7)	134	.54 (1.0)
	Blood levels of glucose	13	5.72 (1.1)	8	5.40 (.9)	74	6.25 (1.1)	102	6.36 (1.5)
	Metabolic syndrome	11	.27 (.5)	8	. 38 (.5)	60	.25 (.4)	99	.38 (.5)
	BMI	37	25.47 (4.3)	33	27.67 (5.9)	174	26.31 (5.7)	267	28.04 (6.7)**
	Number of psychotropic medication	20	.70 (.7)	21	.62 (.6)	97	.64 (.7)	176	.73 (.7)
Smoking	-	38	4.87 (8.7)	30	8.33 (9.3)	166	5.99 (8.1)	252	9.87 (10.8)**
Alcohol use	-	37	2.84 (2.3)	28	3.36 (3.9)	161	3.77 (4.8)	248	3.93 (5.7)
Global disability		39	20.31 (7.0)	33	28.12 (8.4)	181	21.83 (6.5)	275	30.15 (9.0)**
Healthy/unhealthy diet	-	39	.97 (.3)	33	1.03 (.4)	181	1.13 (.6)	274	1.17 (.6)
Psychological distress		36	31.33 (10.7)	32	50.22 (11.0)	169	38.11 (12.5)	260	52.10 (10.8)**

^a^ = sum score of the childhood maltreatment present or absent subtypes (range 0–5)

^+^ = a Chi-square test was conducted

*p* <.001**. No ANOVAs were conducted on the PCL-5 and CTQ-SF subscales. Alcohol use was assessed using the AUDIT, which measures alcohol consumption, drinking behaviours, and alcohol-related problems. Global disability was measured with the WHO-DAS 2.0, indicating the extent to which health issues interfered with daily functioning in the past 30 days. Psychological distress reflects scores on the OQ-45 Symptomatic Distress subscale, capturing symptoms of depression, stress, and anxiety

## Discussion

This explorative study including a sample of ‘real world’ patients referred to secondary psychiatric care aimed to investigate the associations between childhood maltreatment, PTSD, and (components of) MetS, while accounting for demographic and lifestyle related risk factors and to exploratively test whether these associations varied by sex.

To summarize: severity of childhood maltreatment was directly related to increased waist circumference and increased diastolic blood pressure, but was not indirectly associated, through PTSD symptom severity, with any of the MetS components, nor a MetS diagnosis. Further, no statistically significant sex-specific interaction effects were found in the associations between childhood maltreatment severity, PTSD symptom severity, and MetS or its constituent components. Lifestyle-related factors including smoking and unhealthy diet seem to exert larger indirect effects on MetS and its components than PTSD symptom severity. Finally, group differences in blood glucose levels were observed, with higher levels in participants with a history of childhood maltreatment compared to those without.

The prevalence of MetS in our sample (43.7% and 26.2% in men and women, respectively) was notably higher than estimates reported within the same age group in the general population in the Northern Netherlands, particularly among men. Van Zon and colleagues (2020) [46], using data from the Lifelines cohort collected in 2013, reported a MetS prevalence of 13% in men and 8% in women aged 35–39 years. Although percentages may have increased in recent years, the prevalence rates observed in our sample are substantially higher, which may reflect the increased somatic risk burden typically observed in psychiatric populations referred to secondary psychiatric outpatient clinics, potentially due to factors such as psychotropic medication use, lifestyle-related behaviours, psychiatric illness severity and chronic stress [47].

Our findings contribute to the ongoing debate regarding the relationship between childhood maltreatment, PTSD, and MetS. Strikingly, we observed significant associations between childhood maltreatment and waist circumference and diastolic blood pressure, although being distant in time from current states on somatic outcomes. While some studies have reported direct associations between (subtypes of) childhood maltreatment and MetS components [18, 19], findings remain inconsistent [20]. These inconsistencies appear to depend on sample characteristics, such as age, and whether the sample is population-based or clinical. For instance, associations found in younger, non-clinical populations, such as female college students, tend to be weaker [48], whereas stronger associations are observed in older clinical adult samples [49–51]. However, even within these adult clinical samples, some associations lose significance after adjusting for confounders such as lifestyle-related behaviours, psychotropic medication and depressive symptoms [50, 51]. It is well known that long-term impact of childhood maltreatment on psychological and general functioning is highly variable, depending on individual differences in both exacerbating and protective genetic and environmental factors [52, 53], which may either buffer or intensify its effects.

While much of the existing literature on PTSD and MetS has focused on veteran populations, limiting generalizability to broader clinical samples, evidence from non-veteran groups, including psychiatric patients and police officers, also indicates an elevated MetS prevalence in those with PTSD [18, 19]. These associations remained significant even after adjusting for covariates, including lifestyle-related factors. However, these studies did not explicitly assess the influence of childhood maltreatment.

Only Franz and colleagues (2019) examined both childhood maltreatment and PTSD in 337 veterans in their early thirties and found that the associations between childhood interpersonal maltreatment (i.e., psychical abuse, sexual abuse, and/or family violence) and cardiometabolic outcomes were stronger than those observed for PTSD [21]. These findings are in line with our study in which we included both childhood maltreatment, PTSD and lifestyle-related factors, and found that childhood maltreatment as well as lifestyle-related factors, but not PTSD were significantly related to cardiometabolic outcomes. However, Franz and colleagues (2019) [21] observed a significant indirect association of childhood maltreatment through PTSD, a discrepancy with our study that may be explained by their somewhat larger, younger, and more homogeneous sample than our more diverse population. Although we did not examine the exact distribution of clinical-derived primary diagnoses, a previous study on mostly the same MOPHAR dataset showed that the group contains individuals with depressive disorders (26.6%), anxiety disorders (21.8%, including PTSD), personality disorders (14.4%), and bipolar disorders (14.3%) as primary diagnosis at baseline [54]. Additionally, Franz and colleagues (2019) [21] included only one covariate (combat exposure), while our study accounted for a broader range of known demographic and lifestyle related risk factors. 

The current study results suggest that lifestyle factors play a more substantial role in the relationship between childhood maltreatment severity and MetS and its components than PTSD symptom severity. These results emphasize the importance of broadening the scope of research on MetS risk by incorporating additional risk factors beyond PTSD, particularly a history of childhood maltreatment and lifestyle-related factors.

With respect to sex differences: we observed increased prevalence of (components of) MetS in men, which aligns with findings from a European study reporting a higher MetS prevalence in men than in women [23], specifically given our relatively young (premenopausal) sample [24]. However, we did not observe statistically significant sex-specific differences in the associations between childhood maltreatment severity, PTSD symptom severity, and MetS or its components in the current study. This contrasts with findings by Lee and colleagues (2014) [29] amongst 1,234 U.S adults, who found that sexual abuse was associated with increased risk only in women [29], and with several studies in Asian samples that found sex-specific patterns in the relationship between PTSD and MetS [25–27, 29]. Our results are more consistent with those of Wolf and colleagues (2016) [28], who did not find sex-specific differences in the longitudinal associations between PTSD and MetS in a clinical U.S. sample. Several factors may account for the observed discrepancies. Our sample was considerably smaller than the sample of Lee and colleagues (2014) [29], which may have limited our ability to detect subtle sex-specific differences. Additionally, we included somewhat different metabolic risk factors than the previous studies. For example, Lee and colleagues (2014) [29] focused on socioeconomic mediators, whereas we took other demographic and lifestyle-related risk factors into account that may exert different effects on metabolic risk within psychiatric populations. Finally, cultural differences, such as dietary habits between European and non-European populations may also contribute to divergent findings.

Our results showed that participants with presence of a history of childhood maltreatment had higher blood levels of glucose compared to those without such a history. Among the four diagnostic groups (with/without presence of a history of childhood maltreatment and with/without presence of a PTSD), significant overall group differences were observed with respect to multiple demographic and lifestyle related risk factors, including BMI, alcohol use, smoking behaviour, global disability, and psychological distress. Post-hoc comparisons revealed that participants with PTSD—either alone or in combination with childhood maltreatment—showed less favourable outcomes across BMI, smoking, psychological distress and global disability, suggesting that a current presence of PTSD partially drives these group differences, more so than a history of childhood maltreatment. However, given the relatively small sample sizes in the subgroups with PTSD only and without childhood maltreatment nor PTSD, these findings should be interpreted with caution. Hypothetically, a current diagnosis of PTSD may lead to increased symptom severity, which in turn exacerbates stress-related symptoms. These heightened stress levels can contribute to the adoption of unhealthier lifestyle-related behaviours, which may indirectly promote metabolic dysfunction. Due to our limited sample size to conduct such complex interactions, we were currently not able to test these.

 However, we did not find an association between PTSD and MetS or its components, despite these significantly increased lifestyle-related risk factors. This might be explained by low power to detect associations due to the particularly small sample sizes in the between-group comparisons. Another explanation for the non-significant findings may be the relatively young age of our sample. Since the risk for MetS increases with age, it is possible that metabolic dysregulation has not yet fully manifested in this group, thereby attenuating the association between risk factors including PTSD symptom severity and MetS. Finally, the high proportion of patients scoring above the PTSD cut-off likely reflects the high comorbidity of PTSD with other common mental disorders typically seen in specialized mental health care institutions. Given the overlap between PTSD symptoms (e.g., sleep disturbances, concentration problems) and symptoms of other psychiatric diagnoses such as depression and anxiety, the reported PTSD scores might have been inflated. This could have led to an overestimation of both PTSD symptom severity and the number of PTSD cases, particularly considering the use of a self-report measure for PTSD assessment. As a result, this may have obscured true associations between PTSD and MetS.

It is important to also acknowledge the limitations of the current study, which warrant further consideration. First, the cross-sectional nature of the study precludes any definitive statements regarding causality. Second, it is unclear which traumatic incidents were associated with PTSD, which precludes any inferences regarding the impact of trauma type. Furthermore, we utilised a self-report measure to establish PTSD symptom severity and a presence of PTSD, which might have hampered certainty on whether participants met the criteria for an actual diagnosis. However, using the PCL-5 to establish a probable diagnosis of PTSD is well established [55]. Moreover, despite a recently proposed cut-off of 29 for Dutch trauma-exposed individuals [56], we employed a more conservative and commonly used cut-off of 33 [36]. Third, we were unable to examine the distinct associations between the five subtypes of childhood maltreatment and MetS or its constituent components. Additionally, due to the limited number of participants who reported neglect without abuse (*n* = 105) or abuse without neglect (*n* = 34), and the strong correlations between the abuse and neglect indices (*r* = 0.658), we were unable to isolate the unique effects of these combined indices separately. Fourth, although our analyses accounted for the potential mediating role of various factors in the association between childhood maltreatment and metabolic outcomes, we acknowledge that our approach does not constitute a formal mediation analysis. More advanced methods (such as structural equation modelling or causal mediation analysis) could provide deeper insight into the direct and indirect pathways underlying these associations. Future research would benefit from applying these techniques to further disentangle the complex mechanisms at play. Fifth, we were unable to include interaction terms between sex and all known demographic and lifestyle related risk factors of MetS due to sample size constraints. As a result, we limited our moderation analyses to interactions between sex and the primary predictors (severity of childhood maltreatment and PTSD symptom). While this approach allowed us to maintain statistical power and model stability, it may have limited our ability to detect more nuanced sex-specific effects involving lifestyle-related differential risk factors between the sexes. Sixth, given our aim to acquire in-depth insight in the associations between childhood maltreatment, PTSD and MetS, we focused on PTSD as a risk factor of childhood maltreatment. At the same time, we fully acknowledge that childhood maltreatment also impacts the onset and persistence of other mental disorders, including depression and anxiety, which therefore might also have relevance when studying childhood adversity and MetS. Seventh, while our hypotheses were theoretically driven and formulated prior to analyses, the data were derived from an existing dataset not originally intended for this specific research question*.* Finally, the numbers of patients who conducted laboratory measurements were relatively limited, which is likely attributable to the impact of the Covid-19 pandemic.

### Conclusions

In light of these limitations and strengths, the present study suggests potential clinical implications. Interestingly, although the mean age of our sample was relatively young (37.83 years), a large proportion of participants scored beyond the cut-offs for MetS or its constituent components, reflecting increased health risks. Following current policies, general practitioners in the Netherlands screen patients on cardiovascular risk factors starting from 45 years of age on. The findings of this study emphasize the necessity to start screening for cardiovascular risk factors in psychiatric populations at earlier ages.

Further, our findings suggest that while childhood maltreatment may have direct effect on increased waist circumference and increased diastolic blood pressure, part of its influence on metabolic health may operate through modifiable behavioural pathways. As such, in treatment attention should be placed on addressing maladaptive lifestyle behaviours, especially to reduce the risk of MetS among patients receiving mental healthcare, with particular attention paid to those with a history of childhood maltreatment. Due to the limited power of our sample, we were unable to rule out potential sex-specific differences in the influence of the various demographic and lifestyle related risk factors. However, some cautious suggestions can be made based on the descriptive findings. Specifically, we observed that a significantly higher proportion of men had poorer metabolic health and engaged in more problematic alcohol use compared to women. This lifestyle-related factor could be a relevant target for specific interventions in men. To better inform such interventions, future studies should explicitly examine sex-specific differences in somatic risk profiles and the underlying mechanisms. Based on these findings, sex-tailored interventions can be developed and tested for their effectiveness in both men and women.

In sum, our findings contribute to a more nuanced understanding of direct and indirect relationships between childhood maltreatment, PTSD and MetS within a heterogeneous psychiatric population. Future studies should aim to replicate and extend our results in larger, diagnostically diverse, ‘real world’ samples and further clarify the indirect role of PTSD and other risk factors in relation to MetS. Moreover, the use of clinical-administered diagnostic tools is recommended to improve diagnostic specificity by disentangling overlapping symptoms.

## Supplementary Information

Supplementary Material 1.

Supplementary Material 2.

## Data Availability

The datasets generated and/or analysed during the current study are not publicly available due to the absence of consent from the participants for open data, but are available from the corresponding author on reasonable request.

## Abbreviations

PTSD: Posttraumatic Stress Disorder
MetS: Metabolic Syndrome
BMI: Body Mass Index
HDL-cholesterol: High-Density Lipoprotein Cholesterol
ATP-III: Adult Treatment Panel III
CVD: Cardiovascular Disease
WHO: World Health Organization
SPSS: Statistical Package for the Social Sciences
OR: Odds Ratio
HR: Hazard Ratio
CI: Confidence Interval
MOPHAR: Monitoring Outcomes of Psychopharmacotherapy
CTQ-SF: Childhood Trauma Questionnaire-Short Form
PCL-5: PTSD Checklist for the Diagnostic and Statistical Manual of Mental Disorders, 5th edition
AUDIT: Alcohol Use Disorders Identification Test
WHO-DAS 2.0: World Health Organization Disability Assessment Schedule
OQ-45: Outcome Questionnaire-45
