# Supplementary material for: Associations between childhood maltreatment, PTSD and metabolic outcomes in patients with common mental disorders at outpatient clinics in specialized care
Source: BMC Psychiatry. 2025 Oct 10;25:966. doi: 10.1186/s12888-025-07346-6 (PMC12512650; doi:10.1186/s12888-025-07346-6)
Supplement: Supplementary file 1 — Supplementary Material 1. [file 12888_2025_7346_MOESM1_ESM.docx]

**Table 2.**Results of multiple (logistic) regression analyses.

| **Dependent variable** | **Waist circumference^a^** | | | | | | | | | **Metabolic syndrome^b^** | | | | | | | | | | | | | | | | | | | | | |  |
| --- | --- | --- | --- | --- | --- | --- | --- | --- | --- | --- | --- | --- | --- | --- | --- | --- | --- | --- | --- | --- | --- | --- | --- | --- | --- | --- | --- | --- | --- | --- | --- | --- |
|  | **Model 1** adjusted *R^2^ = .*013 | | | | **Model 2** adjusted *R^2^ = .*172 | | | | | **Model 1** Nagelkerke’s *R^2^* = .004 | | | | | | | | | | | | | **Model 2** Nagelkerke’s *R^2^* = .427 | | | | | | | | |  |
|  | **B [95% CI]** | | β | | **B [95% CI]** | | β | | | **B [95% CI]** | | | | **OR [95% CI]** | | | | | | | **B [95% CI]** | | | OR **[95% CI]** | | | | | | | |  |
| **Constant** | 97.639 [96.14, 99.13]** | | | | 104.024 [99.89, 108.15]** | |  | | | -.717 [-1.03, -.40]** | | | | | .488 [.36, .67] | | | | | | .046 [-.84, .93] | | | 1.047 [.43, 2.54] | | | | | | | |  |
| **Childhood maltreatment** | 2.115 [.060, 3.62]** | | .121 | | 1.384 [-1.98, 4.75] | | .084 | | | .119 [-.20, .43] | | | | | 1.126 [.82, 1.54] | | | | | | .223 [-.69, 1.14] | | | 1.250 [.50, 3.13] | | | | | | | |  |
| **PTSD symptom severity** |  | |  | | 2.131 [-2.12, 6.38] | | .121 | | |  | |  | | | |  | | | | | .342 [-.70, 1.38] | | | | 1.407 [.50, 3.98] | | | | | | | |
| **Childhood maltreatment severity x sex** |  | |  | | -.716 [-5.11, 3.68] | | -.033 | | |  | |  | | | |  | | | | | -.198 [-1.41, 1.01] | | | | .821 [.68, 7.60] | | | | | | | |
| **PTSD symptom severity x sex** |  | |  | | .303 [-4.43, 5.04] | | .013 | | |  | |  | | | |  | | | | | 549 [-.071, 1.81] | | | | 1.732 [1.61, 19.85] | | | | | | | |
|  | **Systolic blood pressure^a^** | | | | | | | | | **Diastolic blood pressure^a^** | | | | | | | | | | | | | | | | | | | | | |  |
|  | **Model 1** adjusted *R^2^* = -.001 | | | | **Model 2** adjusted *R^2^ = .*332 | | | | | **Model 1** adjusted *R^2^* = *.*010 | | | | | | | | | | | | | **Model 2** adjusted *R^2^ = .*232 | | | | | | | | |  |
|  | **B [95% CI]** | | β | | **B [95% CI]** | | β | | | **B [95% CI]** | | | | | | | | β | | | | | **B [95% CI]** | | | | | β | | | |  |
| **Constant** | 128.957 [127.37, 130.54]** | | |  | 136.849 [132.85, 140.84]** | |  | | | 82.364 [81.39, 83.34]** | | | | | | | |  | | | | | 86.331[83.93, 88.73]** | | | | |  | | | |  |
| **Childhood maltreatment** | .509 [-1.07, 2.09] | -.028 | | | .220 [-3.04, 3.48] | | .013 | | | 1.201 [0.23, 2.17]* | | | | | | | | .107 | | | | | 1.305 [-.66, 3.26] | | | | | .133 | | | |  |
| **PTSD symptom severity** |  | |  | | .184 [-3.85, 4.21] | | .010 | | |  | |  | | | | | | |  | | | -1.507 [-3.93, .92] | | | | | | | -.100 | | | |
| **Childhood maltreatment severity x sex** |  | |  | | -.806 [-4.99, 3.38] | | -.036 | | |  | |  | | | | | | |  | | | -1.152 [-3.67, 1.36] | | | | | | | -.092 | | | |
| **PTSD symptom severity x sex** |  | |  | | -.270 [-4.78, 4.24] | | -.011 | | |  | |  | | | | | | |  | | | .879 [-1.83, 3.59] | | | | | | | .064 | | | |
|  | **Blood levels of HDL-cholesterol^a^** | | | | | | | | | **Blood levels of triglycerides^a^** | | | | | | | | | | | | | | | | | | | | | |  |
|  | **Model 1** adjusted *R^2^* = -.001 | | | | **Model 2** adjusted *R^2^ = .*169 | | | | | **Model 1** adjusted *R^2^* = *.*006 | | | | | | | | | | | | | **Model 2** adjusted *R^2^ = .*313 | | | | | | | | |  |
|  | **B [95% CI]** | | β | | **B [95% CI]** | | β | | | **B [95% CI]** | | | | | | | | β | | | | | **B [95% CI]** | | | | | β | | | |  |
| **Constant** | 1.342 [1.29, 1.39]** | |  | | 1.276 [1.13, 1.42]** | |  | | | 1.477 [1.37, 1.59]** | | | | | | | |  | | | | | 1.768 [1.44, 2.10]** | | | | |  | | | |  |
| **Childhood maltreatment** | -.024 [-0.07, 0.03] | | -.058 | | -.012 [-.14, .12] | | -.031 | | | .093 [-.02, .021] | | | | | | | | .097 | | | | | .187 [-.11, .48] | | | | | .199 | | | |  |
| **PTSD symptom severity** |  | |  | | -.076 [-.22, .07] | | -.209 | | |  | |  | | | | | | |  | | | .038 [-.29, .36] | | | | | | | .042 | | | |
| **Childhood maltreatment severity x sex** |  | |  | | .080 [-.08, .24] | | .157 | | |  | |  | | | | | | |  | | | -.329 [-.70, .04] | | | | | | | -.259 | | | |
| **PTSD symptom severity x sex** |  | |  | | .009 [-.15, .17] | | .018 | | |  | |  | | | | | | |  | | | .053 [-.31, 0.42] | | | | | | | .041 | | | |
|  | **Blood levels of glucose^a^** | | | | | | | | |  | |  | | | | | | | | | | | | | | | | | | | | |
|  | **Model 1** adjusted *R^2^ =* -.001 | | | | | **Model 2** adjusted *R^2^ = .*125 | | |  | | | | | | | | | | | |  | | | | | |  | | | |  |  |
|  | **B [95% CI]** | | β | | | **B [95% CI]** | | β | | |  | |  | | | | | | | |  | | | | | |  | | | |  |  |
| **Constant** | 6.420 [6.23, 6.61]** | |  | | | 6.582 [6.14, 7.02]** | |  | | |  | | | | | |  | | |  | | | | | |  | | | |  |  |  |
| **Childhood maltreatment** | .096 [-.10, .30] | | .068 | | | -.137 [-.52, .25] | | -.146 | | |  | | | | | |  | | |  | | | | | |  | | | |  |  |  |
| **PTSD symptom severity** |  | |  | | | .083 [-.32, .48] | | .093 | | |  | | | | | |  | | |  | | | | | |  | | | |  |  |  |
| **Childhood maltreatment severity x sex** |  | |  | | | .181 [-.29, .66] | | .138 | | |  | | | | | |  | | |  | | | | | |  | | | |  |  |  |
| **PTSD symptom severity x sex** |  | |  | | | -.290 [-.74, .16] | | -.221 | | |  | | | | | |  | | |  | | | | | |  | | | |  |  |  |

**Note.** Childhood maltreatment severity and PTSD symptom severity were assessed using the CTQ-SF and PCL-5, respectively. In the analyses, men were coded as 0 and women as 1. **B** = unstandardized beta-coefficient; β = standardized beta-coefficient; CI = confidence interval; *p* < .005*, *p*< .001**. All predictors were standardized (z-scores) prior to analysis. The interaction term represents the product of the standardized variables. In this table*,* only (un)standardized beta-coefficients of childhood maltreatment severity, PTSD symptom severity, and their interactions with sex are visualized. However, all known risk factors were included in Models 2. For these results, see Appendix D. **B** = unstandardized beta-coefficient; β = standardized beta-coefficient; OR = odds ratio; ^a^ multiple regression; ^b^ multiple logistic regression.

**Table 3.**

Results of two-way ANOVA analyses.

|  | **Waist circumference** | | **Systolic blood pressure** | | **Diastolic blood pressure** | | **Blood levels of HDL-cholesterol** | | | **Blood levels of triglycerides** | | | **Blood levels of glucose** | |
| --- | --- | --- | --- | --- | --- | --- | --- | --- | --- | --- | --- | --- | --- | --- |
|  | **MD** | ***p*** | **MD** | ***p*** | **MD** | ***p*** | **MD** | ***p*** | | **MD** | | ***p*** | **MD** | ***p*** |
| **PTSD present/absent** | 2.96 | .117 | .08 | .567 | .94 | .336 | .0013 | .961 | | .1913 | | .091 | .1221 | .751 |
| **Childhood maltreatment present/absent** | 1.42 | .652 | 1.99 | .405 | 2.29 | .135 | .1006 | .183 | | .0083 | | .864 | .7224 | .020* |
| **Interaction-effect** |  | .700 |  | .404 |  | .556 |  | .901 |  | |  | .442 |  | .504 |

**Note.** BP = blood pressure; HDL-C = HDL-cholesterol; MD = mean difference; *p* < .005*.

**Table 4.**
Demographic and clinical characteristics per group with/without a history of childhood maltreatment and with/without PTSD.

| **Variable** | **Subscale** | |  | **Mean (SD)** |  | **Mean (SD)** |  | **Mean (SD)** |  | **Mean (SD)** |
| --- | --- | --- | --- | --- | --- | --- | --- | --- | --- | --- |
|  |  | | ***n*** | **No childhood maltreatment, nor PTSD** | ***n*** | **PTSD only** | ***n*** | **Childhood maltreatment only** | ***n*** | **Childhood maltreatment and PTSD** |
|  |  | **Men** | | 19 (48.7%) |  | 14 (42.4%) |  | 87 (48.1%) |  | 108 (39.3%) |
|  |  | **Women** | | 20 (51.3%) |  | 19 (57.6%) |  | 94 (51.9%) |  | 167 (60.7%) |
|  |  | **Age total** | | 36.54 (12.5) |  | 39.18 (13.7) |  | 37.45 (11.8) |  | 38.11 (11.7) |
| **PTSD** |  | | 39 | 18.21 (10.3) | 33 | 45.12 (8.0) | 181 | 20.07 (8.4) | 275 | 48.33 (9.9) |
| **Childhood maltreatment** | Emotional abuse | | 39 | 5.79 (1.0) | 33 | 5.76 (1.1) | 181 | 9.46 (3.9) | 275 | 13.18 (5.7) |
|  | Physical abuse | | 39 | 5.00 (.0) | 33 | 5.06 (.4) | 181 | 6.14 (2.6) | 275 | 7.87 (4.6) |
|  | Sexual abuse | | 39 | 5.00 (.0) | 33 | 5.00 (.0) | 181 | 6.29 (3.1) | 275 | 8.82 (6.1) |
|  | Emotional neglect | | 39 | 7.08 (1.4) | 33 | 6.94 (.2) | 181 | 14.19 (4.2) | 275 | 16.23 (4.8) |
|  | Physical neglect | | 39 | 5.26 (.6) | 33 | 5.18 (.5) | 181 | 7.64 (2.7) | 275 | 9.09 (3.8) |
|  | Severity (0-5)^a^ | | 39 | .00 (.0) | 33 | .00 (.0) | 181 | 2.24 (1.1) | 275 | 3.01 (1.4) |
| **Somatic** | Waist circumference | | 37 | 94.35 (14.1) | 33 | 98.73 (17.0) | 173 | 96.23 (18.0) | 266 | 98.88 (17.3) |
|  | Blood pressure | |  |  |  |  |  |  |  |  |
|  | - Systolic | | 37 | 125.689 (16.3) | 33 | 129 (22.8) | 170 | 129.61 (15.6) | 268 | 128.99 (19.4) |
|  | - Diastolic | | 37 | 79.32 (10.1) | 33 | 81.58 (11.6) | 170 | 82.35 (11.0) | 268 | 82.90 (11.5) |
|  | Blood levels of HDL-cholesterol | | 16 | 1.25 (.3) | 15 | 1.26 (.4) | 93 | 1.36 (.4) | 134 | 1.35 (.4) |
|  | Blood levels of triglycerides | | 16 | 1.28 (.8) | 15 | 1.70 (1.1) | 93 | 1.38 (.7) | 134 | .54 (1.0) |
|  | Blood levels of glucose | | 13 | 5.72 (1.1) | 8 | 5.40 (.9) | 74 | 6.25 (1.1) | 102 | 6.36 (1.5) |
|  | Metabolic syndrome | | 11 | .27 (.5) | 8 | . 38 (.5) | 60 | .25 (.4) | 99 | .38 (.5) |
|  | BMI | | 37 | 25.47 (4.3) | 33 | 27.67 (5.9) | 174 | 26.31 (5.7) | 267 | 28.04 (6.7)** |
|  | Number of psychotropic medication | | 20 | .70 (.7) | 21 | .62 (.6) | 97 | .64 (.7) | 176 | .73 (.7) |
| **Smoking** | - | | 38 | 4.87 (8.7) | 30 | 8.33 (9.3) | 166 | 5.99 (8.1) | 252 | 9.87 (10.8)** |
| **Alcohol use** | - | | 37 | 2.84 (2.3) | 28 | 3.36 (3.9) | 161 | 3.77 (4.8) | 248 | 3.93 (5.7) |
| **Global disability** |  | | 39 | 20.31 (7.0) | 33 | 28.12 (8.4) | 181 | 21.83 (6.5) | 275 | 30.15 (9.0)** |
| **Healthy/unhealthy diet** | - | | 39 | .97 (.3) | 33 | 1.03 (.4) | 181 | 1.13 (.6) | 274 | 1.17 (.6) |
| **Psychological distress** |  | | 36 | 31.33 (10.7) | 32 | 50.22 (11.0) | 169 | 38.11 (12.5) | 260 | 52.10 (10.8)** |

**Note.** ^a^ = sum score of the childhood maltreatment present or absent subtypes (range 0-5); ^+^ = a Chi-square test was conducted.; *p*< .001**. No ANOVAs were conducted on the PCL-5 and CTQ-SF subscales. Alcohol use was assessed using the AUDIT, which measures alcohol consumption, drinking behaviours, and alcohol-related problems. Global disability was measured with the WHO-DAS 2.0, indicating the extent to which health issues interfered with daily functioning in the past 30 days. Psychological distress reflects scores on the OQ-45 Symptomatic Distress subscale, capturing symptoms of depression, stress, and anxiety.
