# Supplementary material for: Associations between childhood maltreatment, PTSD and metabolic outcomes in patients with common mental disorders at outpatient clinics in specialized care
Source: BMC Psychiatry. 2025 Oct 10;25:966. doi: 10.1186/s12888-025-07346-6 (PMC12512650; doi:10.1186/s12888-025-07346-6)
Supplement: Supplementary file 2 — Supplementary Material 2. [file 12888_2025_7346_MOESM2_ESM.docx]

**Appendix A**

Comparison between total sample of people at baseline without/with CTQ-SF and without/with PCL-5.

| **Participants** | **Variable under consideration** | **Proportion** |
| --- | --- | --- |
| **Without available CTQ-SF information at baseline** | Gender | 236 (43.4%) |
|  |  | **Mean value (SD)** |
|  | Age | 37.90 (12.0) |
|  |  | **Beyond cut-off** |
|  | Waist circumference | 162 (51.6%) |
|  | Systolic blood pressure | 159 (50.6%) |
|  | Diastolic blood pressure | 68 (46.9%) |
|  | Blood levels of HDL-cholesterol | 58 (40.0%) |
|  | Blood levels of triglycerides | 83 (9.6%) |
|  | Blood levels of glucose | 389 (38.8%) |
|  | Metabolic syndrome | 185 (67.6%) |
|  |  | **Proportion** |
| **With available CTQ-SF information at baseline** | Gender | 799 (41.5%) |
|  |  | **Mean value (SD)** |
|  | Age | 46.35 (16.8) |
|  |  | **Beyond cut-off** |
|  | Waist circumference | 1087 (61.3%) |
|  | Systolic blood pressure | 840 (61.6%) |
|  | Diastolic blood pressure | 763 (56.0%) |
|  | Blood levels of HDL-cholesterol | 396 (41.3%) |
|  | Blood levels of triglycerides | 361 (37.7%) |
|  | Blood levels of glucose | 14 (6.8%) |
|  | Metabolic syndrome | 60 (32.4%) |
|  |  | **Proportion** |
| **Without available PCL-5 information at baseline** | Gender | 793 (41.5%) |
|  |  | **Mean value (SD)** |
|  | Age | 46.32 (17.0) |
|  |  | **Beyond cut-off** |
|  | Waist circumference | 836 (61.8%) |
|  | Systolic blood pressure | 759 (56.1%) |
|  | Diastolic blood pressure | 391 (41.2%) |
|  | Blood levels of HDL-cholesterol | 358 (37.7%) |
|  | Blood levels of triglycerides | 83 (9.7%) |
|  | Blood levels of glucose | 385 (38.7%) |
|  | Metabolic syndrome | 192 (66.8%) |
|  |  | **Proportion** |
| **With available PCL-5 information at baseline** | Gender | 242 (43.7%) |
|  |  | **Mean value (SD)** |
|  | Age | 38.15 (11.9) |
|  |  | **Beyond cut-off** |
|  | Waist circumference | 293 (54.8%) |
|  | Systolic blood pressure | 166 (51.1%) |
|  | Diastolic blood pressure | 163 (50.2%) |
|  | Blood levels of HDL-cholesterol | 73 (47.7%) |
|  | Blood levels of triglycerides | 61 (39.9%) |
|  | Blood levels of glucose | 14 (6.6%) |
|  | Metabolic syndrome | 64 (33.2%) |

**Appendix B**

*Data cleaning/inspection*

It is probable that the HDL-cholesterol level of 11.0 observed for one participant was a typographical error, as was the case with the BMI of 105.7, which were set to missing. Some participants’ data on blood pressures was designated as missing due to the occurrence of one or more of the following circumstances: a higher diastolic than systolic blood pressure, a blood pressure value of zero or one, or the absence of either systolic or diastolic blood pressure readings. Additionally, waist circumferences of zero were recoded as missing.

*Assumptions*

It was anticipated that independence of observations would be achieved as a result of the study design. Multicollinearity was not considered a problematic issue, as the 12 highest Variance Inflation Factors (VIFs) values out of 96 (12.5%) were not high (1 - 2.3; > 2.3) with a maximum value of 2.72. In the case of the sex-specific models, multicollinearity was not an issue since the highest 23.5% and 7.8% of VIFs were not elevated (1 - 2.3; > 2.3), with highest values observed at 4.23 and 3.19 for men and women, respectively. Furthermore, bivariate Spearman correlations were calculated between all continuous, non-standardized variables for the sample included in the analyses (see Appendix E and Appendix F). Residual plots were inspected for linearity and homoscedasticity of residuals in order to ascertain the suitability of the linear regression analysis. To improve linearity and homoscedasticity, only five and two participants were excluded from the analyses with blood levels of glucose, respectively. The potential influence of outliers and distributions were also considered, despite the a priori expectation of non-normality for some somatic and psychological/behavioural variables. Non-normally distributed variables (physical abuse, sexual abuse, physical neglect, smoking, alcohol use, healthy/unhealthy diet, waist circumference, diastolic blood pressure, blood levels of HDL-cholesterol, blood levels of triglycerides, and blood levels of glucose, and BMI) underwent log, inverse and square root transformations. Notwithstanding some improvements in normality, it was observed that no singly type of transformation yielded satisfactory results for all variables. Consequently, the introduction of multiple transformations into the analysis framework introduced a degree of complexity, potentially complicating the interpretation of the results. Since the optimal transformation for each variable had a negligible impact on the significance of the correlations, and the exclusion of participants whose value was 2.5 times the standard deviation above the mean also yielded unsatisfactory results, no variables underwent transformation and no potential outliers were excluded.

*Model tests*

The following adjusted *R*^2^ values for the multiple regression models, including childhood maltreatment as a predictor (Models 1), were observed: waist circumference (adjusted *R^2^ = .*013, 7.511(1, 507)), systolic blood pressure (adjusted *R^2^* = -.001, .398 (1, 506)), diastolic blood pressure (adjusted *R^2^* = *.*010, 5.887(1, 506)), blood levels of HDL-cholesterol (adjusted *R^2^* = -.001, .867(1, 256)), blood levels of triglycerides (adjusted *R^2^* = *.*006, 2.432 (1, 256)), and blood levels of glucose (adjusted *R^2^ =* -.001, .897(1, 195)). Among these, the models for waist circumference (*p* < .001) and diastolic blood pressure (*p* = .016) reached statistical significance. The model for MetS yielded a Nagelkerke’s *R^2^* value of .004. Sample sizes varied across outcomes, ranging from *n* = 178 for MetS to *n* = 509 for waist circumference.

The following adjusted *R*^2^ values for the multiple regression models, including childhood maltreatment, PTSD, age, sex, lifestyle-related behaviours, psychological distress, BMI and use of psychotropic medication (Models 2), were observed: waist circumference (adjusted *R^2^ = .*172, 4.456(15, 234)), systolic blood pressure (adjusted *R^2^ = .*332, 8.715(16, 232)), diastolic blood pressure (adjusted *R^2^ = .*232, 5.693(16, 232)), blood levels of HDL-cholesterol (adjusted *R^2^ = .*169, 2.346(16, 90)), blood levels of triglycerides (adjusted *R^2^ = .*313, 4.014(16, 90)), and blood levels of glucose (adjusted *R^2^ = .*125, 1.725(16, 65)). Only the model for blood levels of glucose was non-significant (*p* = .064). The model for MetS achieved a Nagelkerke’s *R^2^* value of .427. Sample sizes varied across outcomes, ranging from *n* = 82 for blood levels of glucose to *n* = 250 for waist circumference.

*Summary of significant risk factors*

Healthy/unhealthy diet, use of antihypertensives, age and sex were related to waist circumference. BMI, age and sex were associated with systolic blood pressure and BMI and age with diastolic blood pressure. BMI and sex were related with blood levels of HDL-cholesterol. Smoking, BMI and were associated with blood levels of triglycerides. Statins and age were associated with blood glucose levels. BMI and sex were significantly related to MetS

**Appendix C**

Descriptives on sex, age and continuous PTSD, childhood maltreatment and known risk factors for MetS for comparison between groups of people at baseline without and with information on continuous MetS components.

|  |  | **Mean(SD)** |  |  |  |  |  |  |  |  |  |
| --- | --- | --- | --- | --- | --- | --- | --- | --- | --- | --- | --- |
|  | **Male** | **Age** | **PTSD symptom severity** | **Childhood maltreatment severity** | **BMI** | **Number of psychotropic medication** | **Smoking** | **Alcohol use** | **Global disability** | **Healthy/ unhealthy diet** | **Psycho-logical distress** |
| **Waist circumference** | | | | | | | | | | | |
| **Without** | 8 (42.1%) | 42.16 (12.6) | 33.89 (16.4) | 2.16 (1.5) | 34.25 (8.6) | .55 (.7) | 5.68 (8.1) | 1.39 (2.1) | 25.9 (10.4) | 1.0 (.0) | 46.37 (11.1) |
| **With** | 220 (43.2%) | 37.6 (11.9) | 36.31 (16.8) | 2.34 (1.5) | 27.16 (6.2) | .70 (.7) | 8.15 (9.9) | 3.85 (5.2) | 26.47 (9.0) | 1.14 (.6) | 45.69 (13.8) |
| **Systolic and diastolic blood pressure** | | | | | | | | | | | |
| **Without** | 103 (46.0%) | 37.14 (11.8) | 33.83 (17.1) | 2.21 (1.5) | 26.51 (6.0) | .70 (.7) | 8.03 (11.0) | 3.83 (5.3) | 24.83 (8.5) | 1.13 (.6) | 43.38 (13.3) |
| **With** | 125 (41.1%) | 38.3 (12.0) | 38.00 (16.4) | 2.42 (1.6) | 27.75 (6.4) | .69 (.7) | 8.1 (9.0) | 3.71 (5.0) | 27.63 (9.3) | 1.13 (.6) | 47.52 (13.7) |
| **Blood levels of HDL-cholesterol and triglycerides** | | | | | | | | | | | |
| **Without** | 167 (42.9%) | 37.7 (11.8) | 35.49 (16.6) | 2.25 (1.5) | 26.60 (5.6) | .65 (.7) | 8.24 (11.1) | 3.84 (5.0) | 25.87 (9.0) | 1.14 (.6) | 44.72 (13.6) |
| **With** | 61 (43.9%) | 38.25 (12.1) | 38.26 (17.1) | 2.56 (1.51) | 29.0 (7.5) | .75 (.7) | 7.50 (9.2) | 3.52 (5.4) | 28.1 (9.0) | 1.10 (.5) | 48.64 (13.6) |
| **Blood levels of glucose** | | | | | | | | | | | |
| **Without** | 139 (42.9%) | 38.13 (11.8) | 36.25 (16.9) | 2.27 (1.6) | 26.83 (5.7) | .66 (.7) | 8.27 (10.0) | 3.73 (5.0) | 26.0 (9.0) | 1.16 (.6) | 45.54 (13.9) |
| **With** | 86 (43.7%) | 37.35 (12.0) | 35.66 (16.6) | 2.41 (1.5) | 27.77 (6.7) | .72 (.7) | 7.57 (9.8) | 3.72 (5.1) | 27.00 (9.1) | 1.09 (.6) | 45.67 (13.4) |

**Note**. Childhood maltreatment severity and PTSD symptom severity were assessed using the CTQ-SF and PCL-5, respectively. In these analyses, the number of experiences subtypes of childhood maltreatment (range 0-5) were used. Alcohol use was assessed using the AUDIT, which measures alcohol consumption, drinking behaviours, and alcohol-related problems. Global disability was measured with the WHO-DAS 2.0, indicating the extent to which health issues interfered with daily functioning in the past 30 days. Psychological distress reflects scores on the OQ-45 Symptomatic Distress subscale, capturing symptoms of depression, stress, and anxiety; ^a^ = sum score of the childhood maltreatment present or absent subtypes (range 0-5);samples of systolic and diastolic blood pressure and HDL-cholesterol and triglycerides are combined since these were comprised of the same participants

**Appendix D**

**Table A.**

Results of multiple regression analyses with waist circumference as dependent variable.

| **Dependent variable** | **Waist circumference** | | | |
| --- | --- | --- | --- | --- |
|  | **Model 1** adjusted *R^2^ = .*013 | | **Model 2** adjusted *R^2^ = .*172 | |
|  | **B [95 % CI]** | β | **B [95 % CI]** | β |
| **Constant** | 97.639 [96.14, 99.13]** |  | 104.024 [99.89, 108.15]** |  |
| **Childhood maltreatment** | 2.115 [.060, 3.62]** | .121 | 1.384 [-1.98, 4.75] | .084 |
| **PTSD symptom severity** |  |  | 2.131 [-2.12, 6.38] | .121 |
| **Alcohol use** |  |  | -1.252 [-3.30, .79] | -.079 |
| **Smoking** |  |  | -.330 [-2.66, 2.00] | -.018 |
| **Healthy/unhealthy diet** |  |  | 3.240 [1.01, 5.47]** | .176 |
| **Global disability** |  |  | .458 [-2.22, 3.14] | .027 |
| **Psychological distress** |  |  | -2.099 [-5.32, 1.12] | -.114 |
| **Antidiabetics** |  |  | .882 [-6.59, 8.35] | .014 |
| **Antihypertensives** |  |  | 5.797 [.45, 11.14]* | .139 |
| **Statins** |  |  | -.964 [-8.60, 6.67] | -.106 |
| **Number of psychotropic medication** |  |  | -.653 [-3.45, 2.14] | -.027 |
| **Age** |  |  | 6.523 [3.60, 9.45]** | .289 |
| **Childhood maltreatment severity x sex** |  |  | -.716 [-5.11, 3.68] | -.033 |
| **PTSD symptom severity x sex** |  |  | .303 [-4.43, 5.04] | .013 |
| **Sex** |  |  | -5.652 [-9.91, -1.40]* | -.162 |

**Note.** Childhood maltreatment severity and PTSD symptom severity were assessed using the CTQ-SF and PCL-5, respectively. Alcohol use was assessed using the AUDIT, which measures alcohol consumption, drinking behaviours, and alcohol-related problems. Global disability was measured with the WHO-DAS 2.0, indicating the extent to which health issues interfered with daily functioning in the past 30 days. Psychological distress reflects scores on the OQ-45 Symptomatic Distress subscale, capturing symptoms of depression, stress, and anxiety. In the analyses, men were coded as 0 and women as 1. **B** = unstandardized beta-coefficient; β = standardized beta-coefficient; CI = confidence interval; *p* < .005*, *p*< .001**. All predictors were standardized (z-scores) prior to analysis. The interaction term represents the product of the standardized variables.

**Table B.**

Results of multiple regression analyses with systolic blood pressure as dependent variable.

| **Dependent variable** | **Systolic blood pressure** | | | |
| --- | --- | --- | --- | --- |
|  | **Model 1**  adjusted *R^2^* = -.001 | | **Model 2** adjusted *R^2^ = .*332 | |
|  | **B [95 % CI]** | β | **B [95 % CI]** | β |
| **Constant** | 128.957 [127.37, 130.54]** |  | 136.849 [132.85, 140.84]** |  |
| **Childhood maltreatment severity** | .509 [-1.07, 2.09] | -.028 | .220 [-3.04, 3.48] | .013 |
| **PTSD symptom severity** |  |  | .184 [-3.85, 4.21] | .010 |
| **Alcohol use** |  |  | -.443 [-2.59, 1.70] | -.024 |
| **Smoking** |  |  | .973 [-1.28, 3.23] | .048 |
| **Healthy/unhealthy diet** |  |  | 2.029 [-.16, 4.22] | .102 |
| **Global disability** |  |  | -1.162 [-3.75, 1.43] | -.065 |
| **Psychological distress** |  |  | .717 [-2.40, 3.84] | .036 |
| **BMI** |  |  | 6.149 [4.31, 7.99]** | .369 |
| **Antidiabetics** |  |  | -1.671 [-8.90, 5.56] | -.025 |
| **Antihypertensives** |  |  | 3.120 [-2.13, 8.37] | .070 |
| **Statins** |  |  | .328 [-7.07, 7.72] | .005 |
| **Number of psychotropic medication** |  |  | -1.368 [-4.08, 1.35] | -.052 |
| **Age** |  |  | 5.329 [2.47, 8.19]** | .218 |
| **Childhood maltreatment severity x sex** |  |  | -.806 [-4.99, 3.38] | -.036 |
| **PTSD symptom severity x sex** |  |  | -.270 [-4.78, 4.24] | -.011 |
| **Sex** |  |  | -10.754[-14.88, -6.63]** | -.287 |

**Note.** Childhood maltreatment severity and PTSD symptom severity were assessed using the CTQ-SF and PCL-5, respectively. Alcohol use was assessed using the AUDIT, which measures alcohol consumption, drinking behaviours, and alcohol-related problems. Global disability was measured with the WHO-DAS 2.0, indicating the extent to which health issues interfered with daily functioning in the past 30 days. Psychological distress reflects scores on the OQ-45 Symptomatic Distress subscale, capturing symptoms of depression, stress, and anxiety. In the analyses, men were coded as 0 and women as 1. **B** = unstandardized beta-coefficient; β = standardized beta-coefficient; CI = confidence interval; *p* < .005*, *p*< .001**. All predictors were standardized (z-scores) prior to analysis. The interaction term represents the product of the standardized variables.

**Table C.**
Results of multiple regression analyses with diastolic blood pressure as dependent variable.

| **Dependent variable** | **Diastolic blood pressure** | | | |
| --- | --- | --- | --- | --- |
|  | **Model 1**  adjusted *R^2^* = *.*010 | | **Model 2** adjusted *R^2^ = .*232 | |
|  | **B [95 % CI]** | β | **B [95 % CI]** | β |
| **Constant** | 82.364 [81.39, 83.34]** |  | 86.331[83.93, 88.73]** |  |
| **Childhood maltreatment severity** | 1.201 [0.23, 2.17]* | .107 | 1.305 [-.66, 3.26] | .133 |
| **PTSD symptom severity** |  |  | -1.507 [-3.93, .92] | -.100 |
| **Alcohol use** |  |  | .037 [-1.25, 1.33] | .004 |
| **Smoking** |  |  | -.189 [-1.54, 1.17] | -.017 |
| **Healthy/unhealthy diet** |  |  | .674 [-.65, 1.99] | .060 |
| **Global disability** |  |  | .094 [-1.46, 1.65] | .009 |
| **Psychological distress** |  |  | .247 [-1.63, 2.12] | .022 |
| **BMI** |  |  | 3.175 [2.07, 4.28]** | .340 |
| **Antidiabetics** |  |  | -3.452 [-7.80, 0.89] | -.093 |
| **Antihypertensives** |  |  | .874 [-2.28, 4.03] | .035 |
| **Statins** |  |  | .011 [-4.43, 4.46] | .000 |
| **Number of psychotropic medication** |  |  | -1.506[-3.14, .12] | -.102 |
| **Age** |  |  | 4.108[2.39, 5.83] ** | .299 |
| **Childhood maltreatment severity x sex** |  |  | -1.152 [-3.67, 1.36] | -.092 |
| **PTSD symptom severity x sex** |  |  | .879 [-1.83, 3.59] | .064 |
| **Sex** |  |  | -1.083 [-3.56, 1.40] | -.052 |

**Note.** Childhood maltreatment severity and PTSD symptom severity were assessed using the CTQ-SF and PCL-5, respectively. Alcohol use was assessed using the AUDIT, which measures alcohol consumption, drinking behaviours, and alcohol-related problems. Global disability was measured with the WHO-DAS 2.0, indicating the extent to which health issues interfered with daily functioning in the past 30 days. Psychological distress reflects scores on the OQ-45 Symptomatic Distress subscale, capturing symptoms of depression, stress, and anxiety. n the analyses, men were coded as 0 and women as 1. **B** = unstandardized beta-coefficient; β = standardized beta-coefficient; CI = confidence interval; *p* < .005*, *p*< .001**. All predictors were standardized (z-scores) prior to analysis. The interaction term represents the product of the standardized variables.

**Table D.**

Results of multiple regression with blood levels of HDL-cholesterol as dependent variable.

| **Dependent variable** | **Blood levels of HDL-cholesterol** | | | |
| --- | --- | --- | --- | --- |
|  | **Model 1**  adjusted *R^2^* = -.001 | | **Model 2** adjusted *R^2^ = .*169 | |
|  | **B [95 % CI]** | β | **B [95 % CI]** | β |
| **Constant** | 1.342 [1.29, 1.39]** |  | 1.276 [1.13, 1.42]** |  |
| **Childhood maltreatment severity** | -.024 [-0.07, 0.03] | -.058 | -.012 [-.14, .12] | -.031 |
| **PTSD symptom severity** |  |  | -.076 [-.22, .07] | -.209 |
| **Alcohol use** |  |  | -.037 [-.10, .03] | .114 |
| **Smoking** |  |  | -.052 [-.13, .03] | -.130 |
| **Healthy/unhealthy diet** |  |  | -.047 [-.13, .03] | -.114 |
| **Global disability** |  |  | -.042 [-.14, .06] | -.115 |
| **Psychological distress** |  |  | .083 [-.03, .19] | .212 |
| **BMI** |  |  | -.061 [-.12, -.01]* | -.215 |
| **Antidiabetics** |  |  | -.026 [-.30, .25] | -.020 |
| **Antihypertensives** |  |  | -.095 [-.29, .10] | -.108 |
| **Statins** |  |  | -.075 [-.33, .18] | -.059 |
| **Number of psychotropic medication** |  |  | -.081 [-.18, .01] | -.157 |
| **Age** |  |  | .060 [-.04, .16] | .124 |
| **Childhood maltreatment severity x sex** |  |  | .080 [-.08, .24] | .157 |
| **PTSD symptom severity x sex** |  |  | .009 [-.15, .17] | .018 |
| **Sex** |  |  | .278 [.14, .42]** | .375 |

**Note.** Childhood maltreatment severity and PTSD symptom severity were assessed using the CTQ-SF and PCL-5, respectively. Alcohol use was assessed using the AUDIT, which measures alcohol consumption, drinking behaviours, and alcohol-related problems. Global disability was measured with the WHO-DAS 2.0, indicating the extent to which health issues interfered with daily functioning in the past 30 days. Psychological distress reflects scores on the OQ-45 Symptomatic Distress subscale, capturing symptoms of depression, stress, and anxiety. In the analyses, men were coded as 0 and women as 1. **B** = unstandardized beta-coefficient; β = standardized beta-coefficient; CI = confidence interval; *p* < .005*, *p*< .001**. All predictors were standardized (z-scores) prior to analysis. The interaction term represents the product of the standardized variables.

**Table E.**

Results of multiple regression analyses with blood levels of triglycerides as dependent variable.

| **Dependent variable** | **Blood levels of triglycerides** | | | |
| --- | --- | --- | --- | --- |
|  | **Model 1**  adjusted *R^2^* = *.*006 | | **Model 2** adjusted *R^2^ = .*313 | |
|  | **B [95 % CI]** | β | **B [95 % CI]** | β |
| **Constant** | 1.477 [1.37, 1.59]** |  | 1.768 [1.44, 2.10]** |  |
| **Childhood maltreatment severity** | .093 [-.02, .021] | .097 | .187 [-.11, .48] | .199 |
| **PTSD symptom severity** |  |  | .038 [-.29, .36] | .042 |
| **Alcohol use** |  |  | -.048 [-.20, .10] | -.060 |
| **Smoking** |  |  | .309 [.13, .49]** | .310 |
| **Healthy/unhealthy diet** |  |  | -.051 [-.23, .13] | -.049 |
| **Global disability** |  |  | -.091 [-.31, .13] | -.100 |
| **Psychological distress** |  |  | -.039 [-.29, .21] | -.040 |
| **BMI** |  |  | .225 [.10, .35]** | .319 |
| **Antidiabetics** |  |  | .142 [-.48, .76] | .043 |
| **Antihypertensives** |  |  | .287 [-.16, .73] | .131 |
| **Statins** |  |  | .385 [-.19, .96] | .123 |
| **Number of psychotropic medication** |  |  | .072 [-.14, .29] | .057 |
| **Age** |  |  | .099 [-.12, .32] | .083 |
| **Childhood maltreatment severity x sex** |  |  | -.329 [-.70, .04] | -.259 |
| **PTSD symptom severity x sex** |  |  | .053 [-.31, 0.42] | .041 |
| **Sex** |  |  | -.475 [-.79, -.16]** | -.258 |

**Note.** Childhood maltreatment severity and PTSD symptom severity were assessed using the CTQ-SF and PCL-5, respectively. Alcohol use was assessed using the AUDIT, which measures alcohol consumption, drinking behaviours, and alcohol-related problems. Global disability was measured with the WHO-DAS 2.0, indicating the extent to which health issues interfered with daily functioning in the past 30 days. Psychological distress reflects scores on the OQ-45 Symptomatic Distress subscale, capturing symptoms of depression, stress, and anxiety. In the analyses, men were coded as 0 and women as 1. **B** = unstandardized beta-coefficient; β = standardized beta-coefficient; CI = confidence interval; *p* < .005*, *p*< .001**. All predictors were standardized (z-scores) prior to analysis. The interaction term represents the product of the standardized variables.

**Table F.**

Results of multiple regression analyses with blood levels of glucose as dependent variable.

| **Dependent variable** | **Blood levels of glucose** | | | |
| --- | --- | --- | --- | --- |
|  | **Model 1**  adjusted *R^2^ =* -.001 | | **Model 2** adjusted *R^2^ = .*125 | |
|  | **B [95 % CI]** | β | **B [95 % CI]** | β |
| **Constant** | 6.420 [6.23, 6.61]** |  | 6.582 [6.14, 7.02]** |  |
| **Childhood maltreatment severity** | .096 [-.10, .30] | .068 | -.137 [-.52, .25] | -.146 |
| **PTSD symptom severity** |  |  | .083 [-.32, .48] | .093 |
| **Alcohol use** |  |  | -.097 [-.31, .11] | -.106 |
| **Smoking** |  |  | -.017 [-.26, .23] | -.016 |
| **Healthy/unhealthy diet** |  |  | -.013 [-.25, .23] | -.012 |
| **Global disability** |  |  | -.125 [-.39, .14] | -.149 |
| **Psychological distress** |  |  | .023 [-.28, .33] | .024 |
| **BMI** |  |  | .068 [-.10, .24] | .092 |
| **Antidiabetics** |  |  | -.434 [-1.52, .65] | -.090 |
| **Antihypertensives** |  |  | -.527 [-1.10, .04] | -.242 |
| **Statins** |  |  | .886 [.06, 1.71]* | .235 |
| **Number of psychotropic medication** |  |  | .124 [-.14, .39] | .099 |
| **Age** |  |  | .474 [.18, .77]** | .391 |
| **Childhood maltreatment severity x sex** |  |  | .181 [-.29, .66] | .138 |
| **PTSD symptom severity x sex** |  |  | -.290 [-.74, .16] | -.221 |
| **Sex** |  |  | -.180 [-.60, .24] | -.098 |

**Note.** Childhood maltreatment severity and PTSD symptom severity were assessed using the CTQ-SF and PCL-5, respectively. Alcohol use was assessed using the AUDIT, which measures alcohol consumption, drinking behaviours, and alcohol-related problems. Global disability was measured with the WHO-DAS 2.0, indicating the extent to which health issues interfered with daily functioning in the past 30 days. Psychological distress reflects scores on the OQ-45 Symptomatic Distress subscale, capturing symptoms of depression, stress, and anxiety. In the analyses, men were coded as 0 and women as 1. **B** = unstandardized beta-coefficient; β = standardized beta-coefficient; CI = confidence interval; *p* < .005*, *p*< .001**. All predictors were standardized (z-scores) prior to analysis. The interaction term represents the product of the standardized variables.

**Table G.**

Results of multiple regression analyses with presence/absence of MetS as dependent variable.

| **Dependent variable** | **Metabolic syndrome** | | | |
| --- | --- | --- | --- | --- |
|  | **Model 1**  Nagelkerke’s *R^2^* = .004 | | **Model 2** Nagelkerke’s *R^2^* = .427 | |
|  | **B [95 % CI]** | **Odds ratio [95 % CI]** | **B [95 % CI]** | **Odds ratio [95 % CI]** |
| **Constant** | -.717 [-1.03, -.40]** | .488 [.36, .67] | .046 [-.84, .93] | 1.047 [.43, 2.54] |
| **Childhood maltreatment severity** | .119 [-.20, .43] | 1.126 [.82, 1.54] | .223 [-.69, 1.14] | 1.250 [.50, 3.13] |
| **PTSD symptom severity** |  |  | .342 [-.70, 1.38] | 1.407 [.50, 3.98] |
| **Alcohol use** |  |  | -.098 [-.54, .34] | .907 [.58, 1.41] |
| **Smoking** |  |  | .318 [-.26, .90] | 1.375 [.77, 2.46] |
| **Healthy/unhealthy diet** |  |  | .139 [-.44, .71] | 1.150 [.65, 2.04] |
| **Global disability** |  |  | -.280 [-.90, .34] | .756 [.41, 1.41] |
| **Psychological distress** |  |  | -.389 [-1.21, .43] | .677 [.30, 1.54] |
| **BMI** |  |  | 1.031 [.48, 1.58]** | 2.804 [1.62, 4.86]** |
| **Number of psychotropic medication** |  |  | .464 [-.22, 1.15] | 1.590 [.80, 3.15] |
| **Age** |  |  | .765 [.10, 1.43]* | 2.148 [1.10, 4.19]* |
| **Childhood maltreatment severity x sex** |  |  | -.198 [-1.41, 1.01] | .821 [.68, 7.60] |
| **PTSD symptom severity x sex** |  |  | 549 [-.071, 1.81] | 1.732 [1.61, 19.85] |
| **Sex** |  |  | -1.431 [-2.52, -.35] * | .239 [.08, 0.71]* |

**Note.** Childhood maltreatment severity and PTSD symptom severity were assessed using the CTQ-SF and PCL-5, respectively. Alcohol use was assessed using the AUDIT, which measures alcohol consumption, drinking behaviours, and alcohol-related problems. Global disability was measured with the WHO-DAS 2.0, indicating the extent to which health issues interfered with daily functioning in the past 30 days. Psychological distress reflects scores on the OQ-45 Symptomatic Distress subscale, capturing symptoms of depression, stress, and anxiety. In the analyses, men were coded as 0 and women as 1. **B** = unstandardized beta-coefficient; β = standardized beta-coefficient; CI = confidence interval; *p* < .005*, *p*< .001**. All predictors were standardized (z-scores) prior to analysis. The interaction term represents the product of the standardized variables.

**Appendix E**

**Table.**
Bivariate Spearman correlations between continuous non-standardized outcome variables, dependent variables and risk factors.

|  | **Waist circumference** | **Systolic blood pressure** | **Diastolic blood pressure** | **Blood levels of HDL-cholesterol** | **Blood levels of triglycerides** | **Blood levels of glucose** |
| --- | --- | --- | --- | --- | --- | --- |
| **Childhood maltreatment severity** | .121** | .028 | .107* | -.058 | -.097 | .068 |
| **PTSD symptom severity** | .078 | -.004 | .047 | -,043 | .097 | .054 |
| **Alcohol use** | -.012 | .032 | -.008 | -.027 | 016 | -.024 |
| **Smoking** | .107* | .103* | .106* | -.168* | .188* | .051 |
| **Healthy/unhealthy diet** | .110* | .100* | .072 | -.234** | .071 | .026 |
| **Global disability** | .133* | -.015 | .042 | -.136* | .105 | .056 |
| **Psychological distress** | .068 | .018 | .060 | -.006 | .122 | .079 |
| **BMI** | .833** | .411** | .393** | -.284** | .407** | .133 |
| **Number of psychotropic medication** | -.018 | -.084 | -.092 | -.108 | .074 | -.104 |
| **Age** | .302** | .353** | .395** | .035 | .253** | .291** |

**Note.** Childhood maltreatment severity and PTSD symptom severity were assessed using the CTQ-SF and PCL-5, respectively. Alcohol use was assessed using the AUDIT, which measures alcohol consumption, drinking behaviours, and alcohol-related problems. Global disability was measured with the WHO-DAS 2.0, indicating the extent to which health issues interfered with daily functioning in the past 30 days. Psychological distress reflects scores on the OQ-45 Symptomatic Distress subscale, capturing symptoms of depression, stress, and anxiety. In the analyses, men were coded as 0 and women as 1. *p* < .005*, *p*< .001**.

**Appendix F**

**Table.**
Bivariate Spearman correlations between continuous non-standardized independent variables and risk factors.

|  | **PTSD symptom severity** | **Alcohol use** | **Smoking** | **Healthy/ unhealthy diet** | **Global disability** | **Psychological distress** | **BMI** | ***n*PM** | **Age** |
| --- | --- | --- | --- | --- | --- | --- | --- | --- | --- |
| **Childhood maltreatment severity** | .403** | .068 | .243** | .073 | .234** | .281** | .161** | .070 | .041 |
| **PTSD symptom severity** |  | .048 | .211** | .071 | .546** | .673** | .140** | .073 | .009 |
| **Alcohol use** |  |  | .197** | .161** | -.065 | .147** | -.056 | .027 | -.090 |
| **Smoking** |  |  |  | .171** | .123** | .152** | .115* | .026 | .105* |
| **Healthy/unhealthy diet** |  |  |  |  | .089* | .134** | .083 | .039 | -.110* |
| **Global disability** |  |  |  |  |  | .625** | .179** | .059 | -.018 |
| **Psychological distress** |  |  |  |  |  |  | .110* | .064 | -.015 |
| **BMI** |  |  |  |  |  |  |  | .016 | .188** |
| ***n*PM** |  |  |  |  |  |  |  |  | .047 |

**Note.** Childhood maltreatment severity and PTSD symptom severity were assessed using the CTQ-SF and PCL-5, respectively. Alcohol use was assessed using the AUDIT, which measures alcohol consumption, drinking behaviours, and alcohol-related problems. Global disability was measured with the WHO-DAS 2.0, indicating the extent to which health issues interfered with daily functioning in the past 30 days. Psychological distress reflects scores on the OQ-45 Symptomatic Distress subscale, capturing symptoms of depression, stress, and anxiety. In the analyses, men were coded as 0 and women as 1. NoPM = Number of psychotropic medication. *p* < .005*, *p*< .001**.
